# Road related pollutants induced DNA damage in dragonfly nymphs (Odonata, Anisoptera) living in highway sedimentation ponds

**DOI:** 10.1038/s41598-019-52207-4

**Published:** 2019-11-05

**Authors:** Sondre Meland, Tânia Gomes, Karina Petersen, Johnny Håll, Espen Lund, Alfhild Kringstad, Merete Grung

**Affiliations:** 1 Norwegian Institute for Water Research (NIVA), Gaustadalléen 21, 0349 Oslo, Norway; 2 Norwegian University of Life Sciences (NMBU), Faculty of Environmental Sciences and Natural Resource Management, PO 5003, 1432 Ås, Norway

**Keywords:** Urban ecology, Environmental impact

## Abstract

Nowadays, stormwater sedimentation ponds are popular in stormwater management because of their ability to mitigate flooding and treat polluted runoff from e.g. roads. In addition, they may provide other ecosystem services such as biodiversity. These man-made habitats will inevitably be polluted and the organisms living therein may be negatively affected by the chemical cocktail present in both the water and sediment compartments. The present study explored DNA damage in dragonfly nymphs (Odonata, Anisoptera) living in highway sedimentation ponds in comparison with natural ponds. The concentrations of Polycyclic aromatic hydrocarbons (PAHs), alkylated PAHs and metals were also determined in sediment samples from the different ponds. The results showed that DNA damage was significantly higher in dragonfly nymphs living in sedimentation ponds compared to nymphs living in natural ponds. DNA damage was also highly and significantly correlated with the pollution levels in the sediment, i.e., PAH and Zinc. Finally, we report the concentrations of various alkylated PAHs which appeared to be very dominant in the sedimentation ponds. Our results show that there may be a conflict between the sedimentation ponds’ primary function of protecting natural water bodies from polluted runoff and their secondary function as habitats for organisms. Overall, we suggest that this must be considered when planning and designing stormwater measures.

## Introduction

Road transportation, transporting humans and goods, are essential in day to day modern life. For example, European economy relies heavily on road transport, with road goods and car passengers accounting for 46% and 73% of intra-EU transport, respectively^1^. Subsequently, roads and road transportation have a huge impact on the environment in terms of land use, causing habitat fragmentation and habitat loss, spreading of invasive organisms, noise, CO_2_ emissions, emissions of particles and a plethora of chemicals polluting local air, soil and water bodies^2^.

Typical examples of pollutants from roads and traffic are particles, nutrients (nitrogen (N) and phosphorus (P)), metals (e.g. copper (Cu), lead (Pb), nickel (Ni), zinc (Zn)) and metalloids (arsenic (As), antimony (Sb)), road salt (sodium chloride (NaCl)) and polycyclic aromatic hydrocarbons (PAHs)^3–5^. These road and traffic related pollutants are readily washed off from the road surface to the surrounding environment during storm events and may finally end up in the aquatic environment, being potentially detrimental to aquatic organisms^6–8^. Hence, polluted road runoff is now acknowledged by most national road administrations (NRAs) and environmental authorities as a significant source of diffuse pollution, and mitigation measures are therefore normally part of road building schemes^9^.

Several mitigation measures, typically phrased under the terms Sustainable Urban Drainage Systems (SUDS) and Best Management Practice (BMP), exist to protect the aquatic environment from polluted road runoff^9^. One of the most popular SUDS is wet sedimentation ponds. These ponds, having permanent water (opposed to dry detention ponds), mimic natural processes and treat road runoff by retaining particle-bound pollutants through sedimentation processes^10^. In addition, dilution, chemical and biological degradation of pollutants are also important for these types of systems. Well-functioning sedimentation ponds have proved to significantly reduce the concentrations of many pollutants and are therefore recognized as robust and cheap ways of protecting nearby water bodies^11^. Hence, thousands of sedimentation ponds and other SUDS have been built along European major roads, including in Norway^9^.

Sedimentation ponds are built to mimic natural ponds and processes and these artificial ponds can be rapidly colonized by aquatic organisms^12,13^. Several recent studies have indicated that highway sedimentation ponds (and also other stormwater sedimentation ponds) are able to support and promote biodiversity at a local and regional scale^14–18^, even specifically for odonatan diversity^19^. These studies underline the sedimentation ponds’ relevance in an ecological context, as they can act as steppingstones and secure connectivity throughout the surrounding landscape. On the other hand, these sedimentation ponds, including other urban stormwater ponds, may become sink habitats and even ecological traps e.g. Villalobos-Jimenez *et al*.^20^. Ponds may contain high levels of pollutants derived from pollution retention and treatment functions, whose accumulation may have short and long-term adverse effects on organisms e.g. Brand, *et al*.^21^, Snodgrass, *et al*.^22^. For example, Grung, *et al*.^23^, Meland, *et al*.^24^ found that organisms inhabiting sedimentation ponds along roads accumulated metals and PAHs. Additionally, Grung *et al*.^23^ documented elevated levels of CYP1A enzyme (Phase I biotransformation enzyme in liver), DNA strand breaks (in blood cells) and PAH metabolites in the bile of the Eurasian minnow (*Phoxinus phoxinus*) living in a highway sedimentation pond. Additionally, high acute mortality of amphibians in sedimentation ponds receiving tunnel wash water has also been observed^25^. Hence, there is an apparent conflict between these ponds’ primary function, which include mitigating peak runoff volumes and pollution reduction, and their secondary function as facilitators for biodiversity. Significant gains must therefore be made in the understanding of the impact that road runoff and associated chemical pollutant cocktail may have on organisms inhabiting sedimentation ponds.

Several of the compounds generally detected in highway runoff sedimentation ponds (like metals and PAHs) are known to induce DNA-damage through various mechanisms including oxidative stress and formation of DNA adducts, which in turn can lead to DNA strand breaks^26^. One of the frequently used methods to assess primary DNA damage in individual cells is the alkaline version of the Comet assay^27^. This method detects DNA single and double-strand breaks (SSB and DSB), alkali-labile sites (AL sites), apurinic/apyrimidinic/abasic sites (AP sites), DNA-DNA/DNA-protein cross-linking, and SSB-associated with incomplete excision repair sites. By using enzyme treatments, other less severe DNA lesions/damages can also be detected. For instance, the bacterial formamidopyrimidine DNA glycosylase (FPG) can be used to detect oxidized purines. FPG, which is a DNA repair enzyme, removes the altered bases and leaves AP sites which are converted to breaks by an associated AP lyase activity or by the high pH used to unwind the DNA^28^. Hence, this additional step in the comet assay relates DNA damage to oxidative stress mechanisms^29^. The comet assay is a sensitive and versatile genotoxicity assay that detects DNA damages in virtually any eukaryotic cell^28^, and it has been successfully applied to a number of different insects and insect cells (including hemocytes in hemolymph)^30^. However, the level of DNA damage in insects that inhabit natural sites has rarely been evaluated.

The main objective of the present study was to assess whether organisms living in highway sedimentation ponds are subjected to genotoxic effects due to their exposure to traffic related pollutants, with special emphasis on PAHs and the metals cadmium (Cd), Cu, Ni and Zn. This was performed by using the comet assay on hemolymph extracted from dragonfly nymphs (Anisoptera, from the genus *Aeshna*) collected from highway sedimentation ponds and natural ponds. Dragonflies are predators and spend most of their lifetime (months – several years) as nymphs in the aquatic environment before they emerge as adults into the terrestrial environment. Hence, dragonfly species may be suitable as a model to assess the ecotoxicological impact aquatic organisms living in sedimentation ponds may experience.

## Results

### Pollution levels

**Figure 1:**
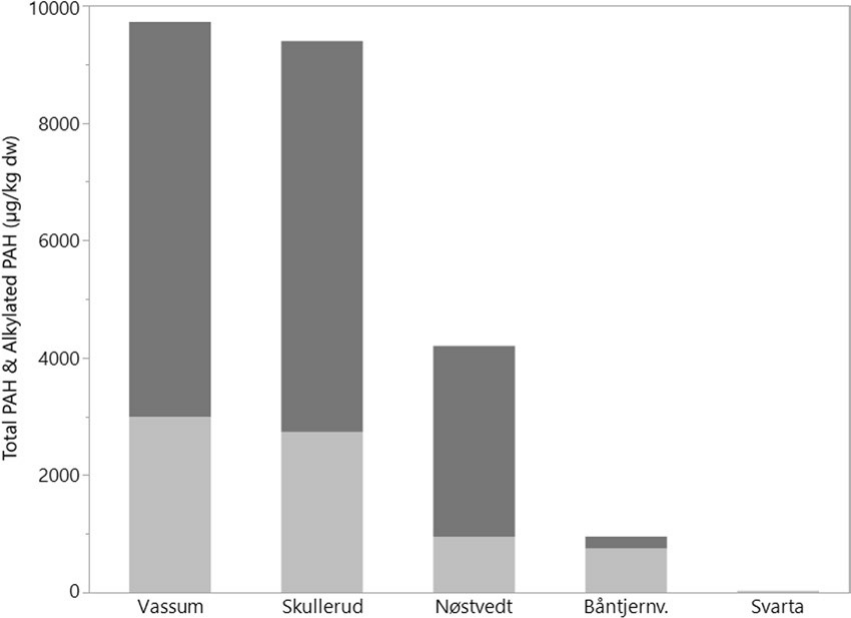
Bar chart showing the concentration (µg/kg dw) of total PAHs (grey) and total alkylated PAHs (dark grey) in the ponds. The concentrations are based on one composite sample.

Compared to the natural ponds, the levels of PAHs and alkylated PAHs were considerable higher in the sedimentation ponds (Fig. 1 and Table 1). Vassum and Skullerud had similar levels, with total concentrations above 9 000 µg/kg dry weight (dw). Nøstvedt had less than half of the concentrations found in Vassum and Skullerud, but still significantly higher than the natural ponds Svarta and Båntjernveien.

**Table 1:** Concentration (µg/kg dw) of PAHs and alkylated PAHs in sediment from the various ponds.

Pond type	Sedimentation pond (µg/kg dw.)			Natural pond (µg/kg dw.)	
Pond	Vassum	Skullerud	Nøstvedt	Båntjernv.	Svarta
Acenaphtene	<40	48 ^AA^	<15	<10	<6
Acenaphthylene	<15	19 ^AA^	<15	<20	<5
Anthracene	41^MAC^	29^MAC^	12^MAC^	11^MAC^	<1
Benzo(a)anthracene*	67^MAC^	98^MAC^	21^AA^	13^AA^	1.7
Benzo(a)pyrene	108^AA^	115^AA^	40^AA^	17^AA^	2.0
Benzo(b,j)fluoranthene*^,#^	221^AA^	220 ^AA^	88	42	4.9
Benzo(e)pyrene	345	285	125	20	2.2
Benzo(ghi)perylene*	235^AA^	84^AA^	117^AA^	<20	<1
Benzo(k)fluoranthene*	54	50	18	7	1.8
Chrysene1	118	175	62	22	2.4
Dibenz(ac/ah)anthracene*	<40	39^MAC^	<15	<8	<1
Dibenzothiophene	19	12	<4	33	<1
Fluoranthene	415^AA^	325	107	333	4.2
Fluorene	64^AA^	35^AA^	18^AA^	29^AA^	<1
Indeno(1,2,3-cd)pyrene*	78^AA^	109^AA^	35	<20	2.1
Naphthalene	47^MAC^	130^MAC^	<15	<20	<10
Perylene	74	125	23	n.a.	<5
Phenanthrene	283^AA^	180^AA^	69^AA^	95^AA^	2.5
Pyrene	825^MAC^	660^MAC^	214 ^MAC^	130^MAC^	3.3
Total PAH	2994	2736	949	752	27
Total PAH16	2556^MAC^	2314^MAC^	801^AA^	699^AA^	25
C1- Dibenzothiophene	101	81	16	18	<10
C1- Naphthalene	45	110	19	<20	<10
C1-Phenanthrene	425	300	67	46	<10
C2- Dibenzothiophene	690	685	142	32	<10
C2- Naphthalene	287	260	386	106	<60
C2- Phenanthrene	1570	1350	356	<60	<10
C3- Dibenzothiophene	1550	1920	372	<60	<10
C3- Naphthalene	1010	975	1660	<100	<100
C3- Phenanthrene	1050	980	234	<60	<10
Total Alkylated PAH	6728	6661	3252	202	n.a.
Total PAH + Alkylated PAH	9722	9396	4201	954	27

Superscript AA indicates concentration above AA-EQS, and superscript MAC indicates concentration above MAC-EQS. EQS derived from the Norwegian classification system of water bodies as part of Norway’s implementation of the EU Water Framework Directive^62^. *PAH with possible carcinogenic properties^63^. ^#^Based on EQS for the benzo(b)fluoranthene compound.

The inclusion of alkylated PAHs substantially raised the levels of PAHs in the sedimentation ponds. The alkylated PAHs summed up to 69%, 71% and 77% of the sum of PAH and alkylated PAH concentrations in Vassum, Skullerud and Nøstvedt, respectively. In contrast, the alkylated PAHs summed up to only 21% in Båntjernveien and were below limit of detection (LOD) in Svarta.

Several of the PAHs present in the sedimentation ponds exceeded either the annual average environmental quality standard (AA-EQS) (e.g. benzo(a)pyrene, benzo(ghi)perylene and fluorene) or the maximum acceptable concentration environmental quality standard (MAC-EQS) (e.g. pyrene, anthracene and benzo(a)anthracene) (Table 1). The natural pond Båntjernveien had also PAHs that exceeded the AA-EQS (e.g. fluorene and benzo(a)pyrene) and MAC-EQS (anthracene only). The PAH concentrations in Svarta were lower and below any EQS.

As for the PAHs, the concentration of metals was in general highest in the sedimentation ponds (Table 2). The concentrations of Ni and Zn in the sedimentation ponds exceeded the AA-EQS and MAC-EQS. The natural pond Båntjernveien appeared to have high concentrations of Cd and Zn, both exceeding the MAC-EQS. The mean concentration of Cd was in fact around twenty times higher in this natural pond compared to the sedimentation ponds.

**Table 2:** Concentration (mg/kg dw) of metals in sediment from the various ponds.

Pond type	Sedimentation pond (mean ± sd (mg/kg dw))			Natural Pond (mean ± sd (mg/kg dw))	
Pond	Vassum (n = 3)	Skullerud (n = 4)	Nøstvedt (n = 3)	Båntjernv. (n = 2)	Svarta (n = 2)
Cadmium	0.18 ± 0.14	0.43 ± 0.2	0.20 ± 0.1	3.75 ± 0.5^MAC^	0.02 ± 0.01
Copper*	130 ± 10	163 ± 36	86.7 ± 56	22.5 ± 0.7	5.40 ± 2.3
Lead*	18.3 ± 2.3	32.0 ± 7.4	18.7 ± 4.9	14.0 ± 1.4	10.1 ± 1.3
Nickel	34 ± 7.0^AA^	41.3 ± 7.5^AA^	32.3 ± 9.9^AA^	28.5 ± 2.1	7.50 ± 6.4
Zinc	780 ± 69^MAC^	648 ± 154^MAC^	410 ± 271^MAC^	375 ± 35^MAC^	15.4 ± 7.9
Sum Metals	963 ± 53	884 ± 203	548 ± 335	444 ± 35	38,4 ± 19

Samples (n) are obtained from previous sampling campaigns in the period 2013–2018. Superscript AA indicates concentration above AA-EQS, and superscript MAC indicates concentration above MAC-EQS. EQS derived from the Norwegian classification system of water bodies as part of Norway’s implementation of the EU Water Framework Directive^62^. * indicates EQS in freshwater sediment, otherwise marine sediment.

The correlations obtained between the metal concentrations and the total concentration of PAHs and alkylated PAHs are displayed in Table 3. The concentrations of Cu, Ni, Pb, Zn and Tot PAHs + alkylated PAHs were strongly correlated, with Pearson correlation coefficients ranging from 0.68 (Zn vs. Pb) to 0.97 (Cu vs. PAHs). In contrast, Cd concentrations were poorly correlated with the other pollutants, in which Pearson correlation coefficients ranged from 0.07 (Cd vs. Ni) to −0.42 (Cd vs. Cu/PAHs).

**Table 3:** Pearson correlation coefficients between mean metal concentrations and total PAH concentrations in sediment from the various ponds (n = 5).

Pollutant	Copper	Nickel	Zinc	Lead	Cadmium	Tot PAH + PAH-alkylated
Copper	1.00					
Nickel	0.85	1.00				
Zinc	0.88	0.89*	1.00			
Lead	0.89*	0.83	0.68	1.00		
Cadmium	−0.42	0.07	−0.07	−0.23	1.00	
Tot PAH+PAH−alkylated	0.97**	0.79	0.92*	0.79	−0.42	1.00

Statistically significant results are indicated by their level of significance: *(p < 0.05) and **(p < 0.01).

### Species identification and nymph size

In Norway, a total of six species belong to the genus *Aeshna* (Norwegian Biodiversity Information Centre, www.biodiversity.no). In the present study, from the sampled nymphs, forty-three (72%) were identified as *Aeshna cyanea*, 14 (23%) were identified as *A*. *juncea*, 2 (3%) nymphs were identified as *A*. *grandis* and 1 nymph (2%) could not be determined down to species (*Aeshna sp*.) (Supplementary Table S1).

The nymph size within each pond was quite similar, but there was a tendency for smaller nymphs in the sedimentation ponds than in the natural ponds. The biggest statistically significant difference in length was obtained between nymphs from Vassum (2.7 ± 0.4 cm) and Svarta (3.2 ± 0.5 cm) (Supplementary Fig. S1).

### DNA damage in Aeshna sp

The DNA damage related to strand breaks and apurinic/apyrimidinic sites (AP sites)/alkali-labile sites (AL sites), as well as less harmful DNA lesions (e.g. DNA adducts and base alterations), of dragonfly nymphs collected from the natural and sedimentation ponds were assessed using the standard comet assay combined with the bacterial repair enzyme FPG. Hydrogen peroxide (H_2_O_2_) was used as a positive control and the obtained results showed a significant increase in DNA damage in dragonfly nymphs, thus assuring a good quality control of the assay (Supplementary Fig. S4). Cell viability was assessed using the trypan blue staining with cell viability > 80% (Supplementary Fig. S5). Images of examples of comets from hemolymph cells from dragonfly nymphs showing different degrees of damage are shown in Supplementary Fig. S6.

**Figure 2:**
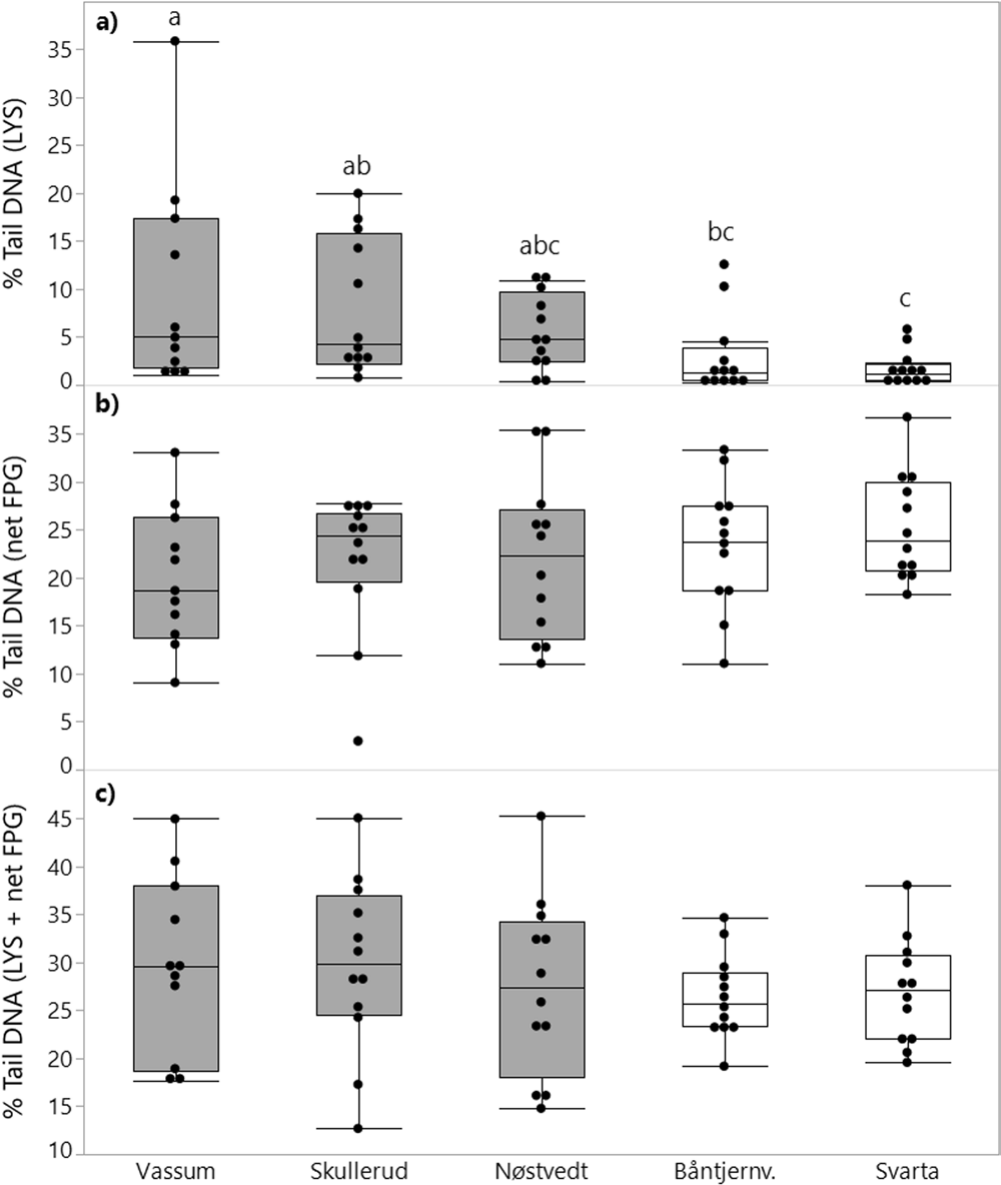
Box-plot showing DNA strand breaks measured in dragonfly nymphs (*Aeshna sp*.) living in highway sedimentation ponds (grey) and natural ponds (white). Individual measurements are included as black points (n = 11–12). (**a**) % Tail DNA (LYS), (**b**) % Tail DNA (net FPG) and (**c**) % Tail DNA (LYS + net FPG). Box-plots with different letters indicate statistically significant differences in DNA strand breaks in nymphs from different ponds (the one-way ANOVA on % Tail DNA (LYS) was performed on log-transformed data). Descriptive statistics of the DNA damage data is also presented in Supplementary Table S2.

In regard to DNA damage linked to strand breaks and AL sites and AP sites (% Tail DNA (LYS)), there were significant differences between nymphs from the different ponds (p = 0.0012, Fig. 2a). The highest DNA damage was observed in nymphs from the Vassum sedimentation pond, followed by Skullerud and Nøstvedt. Percent Tail DNA (LYS) in nymphs from Nøstvedt was, however, not statistically different from % Tail DNA (LYS) in nymphs living in the natural ponds.

The inclusion of net FPG sensitive sites increased the sensitivity of the comet assay (Fig. 2b), and the recorded DNA damage was substantially higher in nymphs from all ponds when compared to the % Tail DNA (LYS). The biggest increase was observed in nymphs from the natural ponds. The ratios of mean % Tail DNA (net FPG) and mean % Tail DNA (LYS) in nymphs from the different ponds were 2.1, 2.7, 4, 7.8 and 14.9 in nymphs from Vassum, Skullerud, Nøstvedt, Båntjernveien and Svarta, respectively. It is a weak tendency, although not statistically significant (*p* = 0.479), that % Tail DNA (net FPG) is slightly higher in nymphs from the natural ponds compared to nymphs from the sedimentation ponds. Likewise, there was no statistical difference in total DNA damage in nymphs from the different ponds measured as % Tail DNA (LYS + net FPG) (p = 0.736, Fig. 2c). However, the variation in % Tail DNA (LYS + net FPG) was apparently bigger in nymphs from the sedimentation ponds compared to nymphs from the natural ponds.

**Figure 3:**
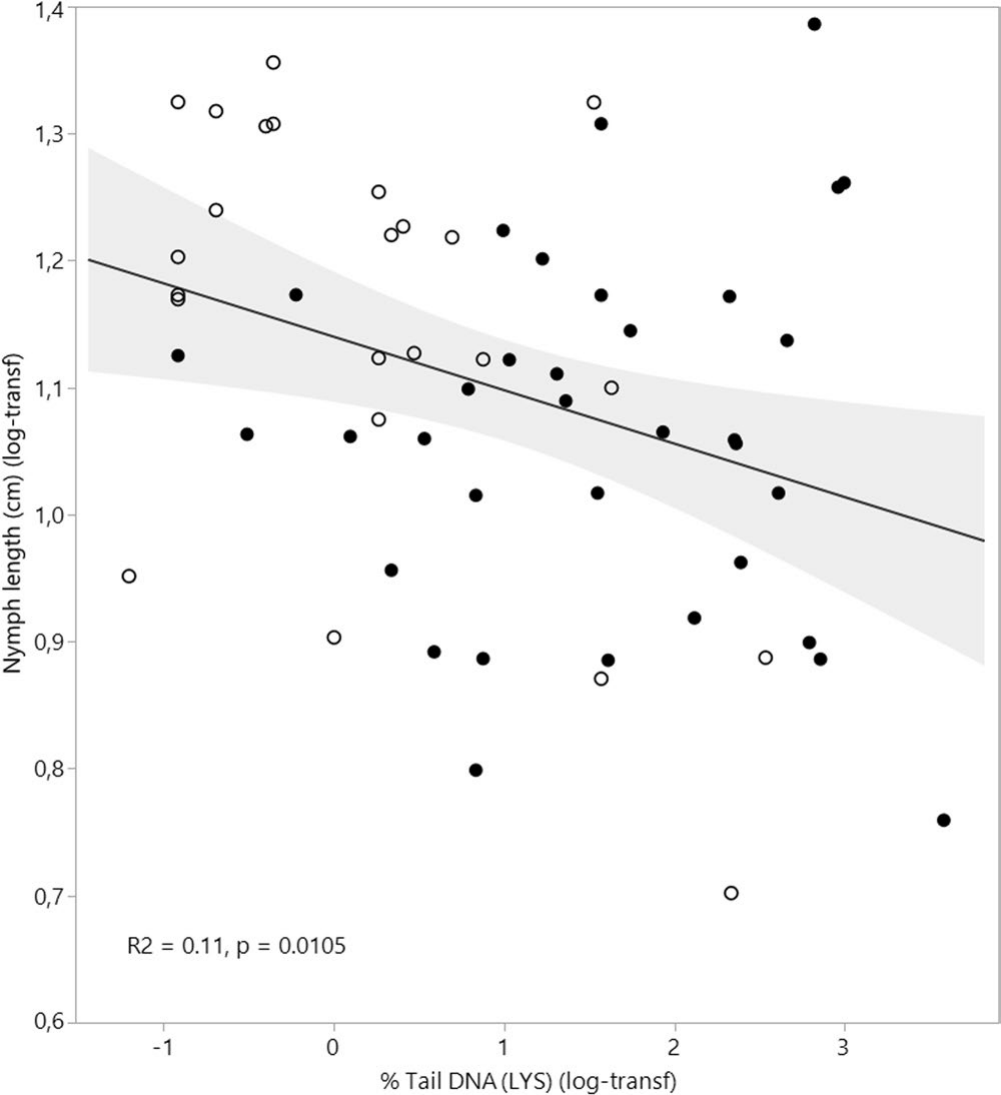
Relationship between DNA damage measured as % Tail DNA (LYS) and size of the dragonfly nymphs measured as length in cm (data log-transformed, n = 59). Nymphs from the natural ponds are indicated by open circles and sedimentation ponds are indicated by black filled circles. Line of fit and 95% confidence intervals are included as solid black lines and grey shading, respectively.

The relationship between DNA damage (LYS, net FPG and LYS + net FPG) and size of the nymphs was explored by permutation based linear regression. Only % Tail DNA (LYS) showed a statistically significant relationship with the nymphs’ size, i.e. a tendency that nymphs having higher levels of DNA damage are shorter than nymphs with less DNA damage. However, the explained variation was weak, only 11% (Fig. 3).

### Relationship between pollution levels and DNA damage in nymphs

**Figure 4:**
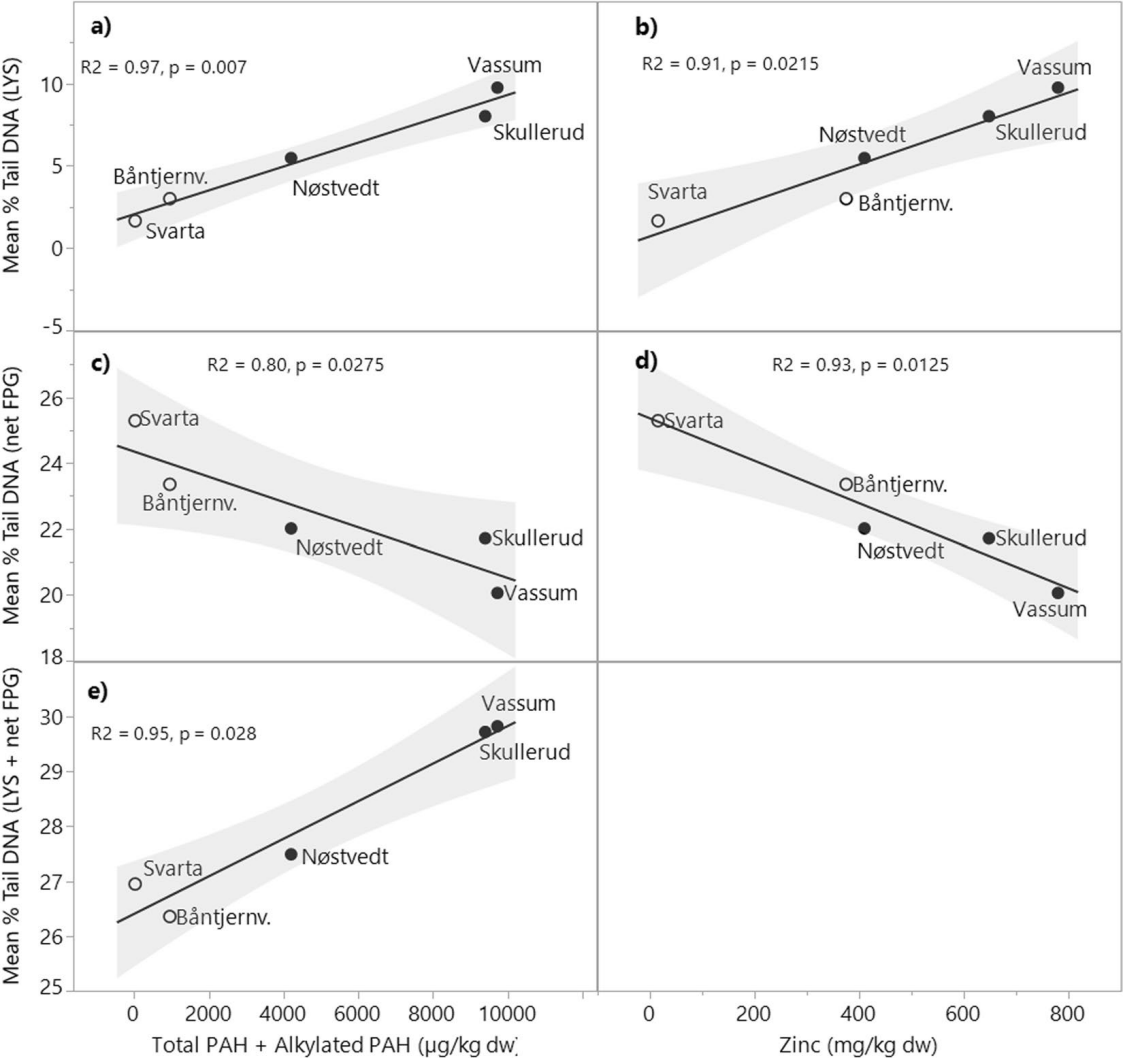
Fitted line plot showing the relationship between the various DNA damage measures (mean values, n = 5. The variation in the data is displayed in Fig. 2) and pollution levels. Only significant results from the linear regression (simple term effects) are displayed. All statistical results are presented in Supplementary Table S3. The plots **a**, **c** and **e** show the relationship between the total concentrations of PAHs and alkylated PAHs in the sediment mean % Tail DNA (LYS), mean % Tail DNA (net FPG) and mean % Tail DNA (LYS + net FPG) in dragonfly nymphs (Aeshna sp.), respectively. The plot **b** and **d** show the relationship between the total concentrations of Zinc in the sediment and mean % Tail DNA (LYS) and mean % Tail DNA (net FPG), respectively. Nymphs from the natural ponds are indicated by open circles and sedimentation ponds are indicated by black filled circles. Line of fit and 95% confidence intervals are included as solid black lines and grey shading, respectively. The X and Y axes have different scales.

The relationship between DNA damage and total PAH concentrations and metals was explored using a permutation based linear regression and multiple regression using RDA. The former statistical approach shows the effect of each single pollutant (simple term effect) on DNA damage whereas the latter picks out the pollutant with the highest effect followed by the pollutant which gives additionally effect (conditional term effect) to the already chosen pollutant(s). The relationship between the length of the nymphs and the pollutants was explored in a similar manner. The statistically significant results from the simple term effects are displayed in Fig. 4, whereas all results are presented in Supplementary Table S3.

As presented in Table 3, the PAH concentrations and metal concentrations, except Cd, were highly correlated. This is also somewhat reflected in the output of the regression analyses on the simple term effects where many of the pollutants showed high correlation with DNA damage (LYS, net FPG and LYS + net FPG), especially total PAH concentrations and Zn and Cu concentrations (Supplementary Table S3). However, only the total PAH concentrations were statistically significant for all three DNA damage measures (Fig. 4a,c,e), while Zn concentrations were significantly correlated with % Tail DNA (LYS) and % Tail DNA (net FPG). In contrast to % Tail DNA (LYS) and % Tail DNA (LYS + net FPG), the correlation between % Tail DNA (net FPG) and the pollutants was negative (Fig. 4c,d). Multiple regression revealed that Total PAH, Zn and Total PAH were significant related to % Tail DNA (LYS), % Tail DNA (net FPG) and % Tail DNA (LYS + net FPG), respectively. None of the pollutants were significantly related to nymph length.

Moran’s I was calculated and statistically tested on the residuals from the statistically significant regression models to disclose any spatial autocorrelation in the data. The output of the Moran’s I displayed a weak (−0.26 to −0.07) but not statistically significant (p > 0.05) dispersion of the data (see Supplementary Table S4). In other words; there was apparently no spatial autocorrelation that biased the regression models.

## Discussion

### Pollution levels in the sediment

The concentrations of PAHs in the sedimentation ponds in the present study are comparable with the highest concentrations found in other Norwegian sedimentation ponds^18,31^. It is also to some extent coherent with a Danish study^11^. However, the present concentrations levels are quite lower than those reported from Canadian studies^32^, which is likely attributed to differences in traffic density.

The inclusion of alkylated PAHs in our study raised the total PAH concentrations in the sediment in all the ponds except the natural pond Svarta. A substantial increase was observed particularly in the sedimentation ponds. Our findings are very coherent with the results obtained in a study of oil sand tailing ponds in Canada^33^. They also observed a similar pattern in both nymphs and imagoes of dragonflies and damselflies, demonstrating a transfer of parental and alkylated PAHs from the surrounding environment to the organisms. Alkylated PAHs had also higher biota-sediment accumulation factors than the parent PAHs. In addition, the high content of alkylated PAHs is a strong indication of petrogenic sources^33,34^. The strong petrogenic signal in our study is slightly contradictory to Richter-Brockmann and Achten^34^ who measured parental PAHs and alkylated PAHs in different environmental matrices including traffic impacted urban soil samples from Germany. In Norway, the use of studded winter tires has a huge impact on road surface wear during winter season. Hence, higher rates of road surface wear (i.e. asphalt and bitumen) might explain this divergence. In addition to road surface wear, the alkylated PAHs may originate from tire wear and potentially oil spill. Excluding the alkylated PAHs, pyrene and fluoranthene were the two dominant PAHs indicating that pyrogenic sources such as combustion are also important. This is in line with other studies, e.g. Istenic, *et al*.^11^. This is the first study, to our knowledge, that has measured alkylated PAHs in highway sedimentation ponds. Our findings suggest that the ponds effectively retain the alkylated PAHs fulfilling their function of protecting water bodies from road runoff. Additionally, our findings suggest that PAH concentrations in sedimentation ponds may have been highly underestimated in previous studies, in particular those related to petrogenic sources. Subsequently, the ecotoxicological risk that organisms inhabiting SUDS face is also underestimated.

Like the PAH concentrations, the highest concentrations of Cu, Pb, Ni and Zn were found in the sedimentation ponds. The AA-EQS and MAC-EQS for Ni and Zn were exceeded in all three sedimentation ponds. The concentrations are comparable to earlier findings in Norway^31^, but generally higher than findings from a Swedish study of 26 sedimentation ponds^35^. Zn is heavily linked to tire wear but is also associated to road and tunnel gear made of galvanized steel, while Cu is associated both to brake wear and tire wear^8^. Surprisingly, the concentrations of both Zn and Cd were high in the natural pond Båntjernveien and exceeded their corresponding MAC-EQS. Elevated concentration levels of several PAHs, Cd and Zn indicate that the pond Båntjernveien is influenced by some diffuse pollution or past chemical spills. The pond is surrounded by residential houses on one of the sides and the pond may have been negatively affected by activity associated to these houses (e.g. car wash). These levels are so high that toxic effects on organisms are to be expected.

### DNA damage in dragonfly nymphs

In this study, an optimization of the standardized Comet Assay protocol has been developed for *Aeshna sp*., by using hydrogen peroxide, a highly recognized genotoxic agent, as positive control. This genotoxicity assay has proven to be a sensitive and reliable tool for the detection of DNA damage in *Aeshna sp*., as previously seen for other insect species^30^.

Revealing genotoxicity by using the versatile and sensitive comet assay is a powerful and cost-efficient tool^36,37^ and has been used in various environmental monitoring studies with various animals from both marine and fresh waters^37^. We have previously documented elevated DNA damage in the Eurasian minnow present in the Skullerud sedimentation pond^23^. However, this is to our knowledge the first time the comet assay is utilized to reveal genotoxicity in insects living in sedimentation ponds or any other SUDS. In addition, DNA damage in dragonfly nymphs has not been documented before. The DNA damage measured as % DNA (LYS) presented a clear and significant indication of genotoxicity in dragonfly nymphs living in the sedimentation ponds. However, the % DNA damage (LYS) in dragonfly nymphs in Skullerud was lower than previously observed in the Eurasian minnow from the same pond (mean % DNA (LYS) = 8% vs 19% and max % DNA (LYS) = 20% vs 53% in nymphs and minnow, respectively)^23^. Genotoxicity using the comet assay was also observed by Haile, *et al*.^26^ on human-derived liver cells, by testing sediments from a road runoff treatment facility in Austria. Authors argued that the observed genotoxicity was linked to PAHs but that combined effects of PAHs and metals could not be disregarded.

PAHs bioaccumulate in organisms and cause DNA damage through metabolic activation^38,39^, mostly through three main pathways; I) formation of dihydro epoxides catalyzed by cytochrome P450 enzymes and epoxide hydrolase which creates stable DNA adducts, II) formation of PAH radical cations in a metabolic oxidation process by cytochrome P450 peroxidase activity generating labile DNA adducts which are eliminated via depurination, resulting in apurinic sites, and III) formation of ortho-quinones via oxidation of catecholes by dihydrodiol dehydrogenase leading to ROS and ROS induced DNA damage. PAH metabolism in insects is not well studied and it is questionable whether the dragonfly nymphs in our study are capable, and to what extent, to biotransform PAHs by cytochrome P450 enzymes. However, Vicentini, *et al*.^40^ showed in an acute exposure study using the chironomid *Chironomus sancticaroli* that benzo(a)pyrene was able to change the metabolic pathway linked to detoxification, nervous system regulation and finally DNA damage. Similarly, the same research group showed that acute and chronic exposure of phenanthrene to the same species caused increased esterase activity (phase I metabolism), histological alterations, DNA damage and reduced growth^41,42^. The length of the nymphs used in the present study was negatively correlated with % Tail DNA (LYS) and a significant difference in length of nymphs living in Vassum and Svarta was evident. Hence, the impact of PAHs on the dragonfly nymphs in the present study might be a significant cause to the observed DNA damage.

Metal genotoxicity occur mainly as an interaction with cellular redox regulation creating ROS and subsequently oxidative DNA damage^29,43^. For example, transition metals such as Iron (Fe) and Cu produce **˙**OH through pathways including Fenton chemistry and Haber-Weiss reactions^44,45^. Cd on the other hand, does not produce free radicals by itself but can replace Cu and Fe in various proteins and thus increasing their share of unbound free metals that can produce ROS through Fenton reactions^43^. In addition, metals may also damage the DNA repair system which may reduce the removal rate of DNA lesions^43^. However, the metal concentrations within the organisms must exceed the organisms’ capacity to produce metal binding proteins in order to have long-term toxicity^46^.

Both metals and PAHs were present at elevated levels in the sediments, especially from the sedimentation ponds, and they were positively correlated with % Tail DNA (LYS). The metal concentrations and the PAH concentrations were also highly correlated. Thus, it is difficult to establish a clear cause and relationship between the observed DNA damage and the two pollutant groups. Interactions between these two pollutant groups may also occur. For example, Fleeger, *et al*.^47^ observed synergistic toxic effects on benthic copepods when exposed to sediment with the PAHs phenanthrene and fluoranthene and the metals Cd, Pb and mercury (Hg). They also showed that the metals acted in an antagonistic way.

Although not measured in the present study, previous studies have demonstrated that metal concentrations in various aquatic insects (including dragonflies from Aeshna) living in sedimentation ponds are higher than in insects living in more natural ponds^24,48^. Meland *et al*.^24^ also found a significant correlation between Zn concentrations in sediment and overall metal burden in the insects and significant correlations between cobalt (Co), Ni, Cd and antimony (Sb) water concentrations and overall insect metal burden. Hence, the elevated levels of metals (except Cd) in the sedimentation ponds compared to the levels in the natural pond may thus be an indication of a corresponding higher level of these metals in the nymphs from the sedimentation ponds compared to the nymphs from the natural ponds. In contrast to % DNA (LYS), the inclusion of net FPG and the sum LYS + net FPG did not reveal any significant differences in nymphs from the various ponds. However, the % increase in DNA damage detected was substantially increased in the natural ponds when including net FPG. The % DNA (LYS) damage is related to single and double strand brakes, while % Tail DNA (net FPG) is associated to less severe DNA damages such as base alterations and base losses^49,50^. Hence, this may be an indication that the frequency of less severe base lesions is higher in nymphs from the natural ponds compared to the nymphs from the sedimentation ponds, and that this may relate to other stressors not determined in this study. For example, temperature, oxygen levels, salinity, stress, diet, reproductive activity and the presence of inhibitors and inducing agents may have a huge impact on ROS generation and enzymes involved in metabolic activation and detoxification^45,51^. The lack of biological endpoints related to ROS production and/or enzymes within the ROS protection machinery (e.g. catalase, superoxide dismutase) is a drawback of our study, and should be included in future studies.

The high levels of Cd in the pond Båntjernveien may be one reason for the DNA damage observed in nymphs from that pond. However, the lack of correlation between Cd and DNA damage suggests that the levels found in Båntjernveien were not high enough to induce damage. In addition, Cd is, according to Nummelin *et al*.^52^, poorly bioaccumulated in dragonfly species such as *A*. *juncea* and *A*. *grandis*, which were present in our study. This is supported by a long-term monitoring study of the export of metals from nine dragonfly and damselfly (Zygoptera) species from the aquatic compartment to the terrestrial compartment where Cd was less exported than e.g. Zn, Cu, Pb and Ni^53^.

Studies have shown that dragonflies can adapt to urbanized landscapes being able to tolerate various stressors^20,54^. This may also be relevant for the dragonflies and other organisms that colonize road sedimentation ponds. However, community studies in highway sedimentation ponds and other stormwater ponds have revealed that pollutant loadings are key drivers for both species richness and community composition. For example, Briers^13^ showed that species richness decreased over time in sedimentation ponds after a quick colonization upon their establishment. Recent Norwegian studies, which also included the sedimentation ponds in the present study, showed that pollution levels in sediments and water had an apparent effect on the ponds’ species composition but not number of taxa^16,18^. More research on whether dragonflies as well as other organisms inhabiting sedimentation ponds are better adapted to resist chronic and acute chemical exposures than those organisms inhabiting natural ponds are warranted.

The sedimentation ponds’ apparent conflict between their primary purpose as efficient treatment systems and their secondary purpose as providers of good habitats for wildlife in the urban environment has been addressed by several authors. For example, Brand, *et al*.^21^ and Snodgrass, *et al*.^22^ argued that road salt, in combination with other pollutants such as metals and PAHs in pond sediments, caused severe damage to three different amphibian species; *Bufo americanus*, *Rana sylvatica and Hyla versicolor*. The emphasis of road salt toxicity was also argued by Bartlett, *et al*.^55^ and Bartlett, *et al*.^32^, who conducted a series of toxicity tests on the amphipod *Hylalella Azteca*. They also concluded that the toxicity observed was more linked to waterborne contaminants rather than contaminants in the sediment. These studies were however based on laboratory experiments. The field approach used in this study seem to support these earlier conclusions. However, our findings may indicate that PAHs and metals in sediment are more significant contributors to toxicity and genotoxicity in sedimentation ponds than previously assessed.

These studies together with our own findings suggest that organisms inhabiting such man-made stormwater habitats may be harmed by the incoming and retained chemical cocktails. The question remains open; what is the tipping point between being a suitable habitat for organisms and/or becoming a sink habitat or even ecological trap? In Norway the public road administration has concluded that nature-based sedimentation ponds should not be used as primary treatment systems for tunnel wash water due to its observed acute toxic effects on amphibians. This is now implemented in their guidelines for design and building of road tunnels^56^. It is urgent that stormwater management balance and weigh the pros and cons when building stormwater ponds. Questions to be raised are for example; 1) what will be the expected pollution levels? 2) in which type of area will the pond be built? 3) will the pond likely be an important provider and contributor to pond connectivity and refuge for organisms? 4) will the ponds contribute to local and/or regional conservation of biodiversity? and 5) how could ponds be designed, built and managed to fulfill their role as water flow and pollution protection measures and providers of wildlife?

To summarize, the comet assay proved to be a suitable tool for studying the genotoxicity in dragonfly nymphs. The DNA damage measured as % Tail DNA (LYS) was significantly higher in dragonfly nymphs living in the sedimentation ponds compared to nymphs living in the natural ponds. This damage was also highly and significantly correlated with the pollution levels in the sediment, i.e. polycyclic aromatic hydrocarbons (PAH) and Zn. The present study shows that there may be a conflict between the sedimentation ponds’ primary function of protecting water bodies from polluted runoff and their secondary function as habitats for organisms. Hence, pros and cons related to the ponds’ ability to mitigate peak runoff volumes, to protect water bodies from pollution and its possibility to be a suitable habitat for wildlife must be considered when planning and designing stormwater measures.

## Materials and Methods

### Study sites

**Figure 5:**
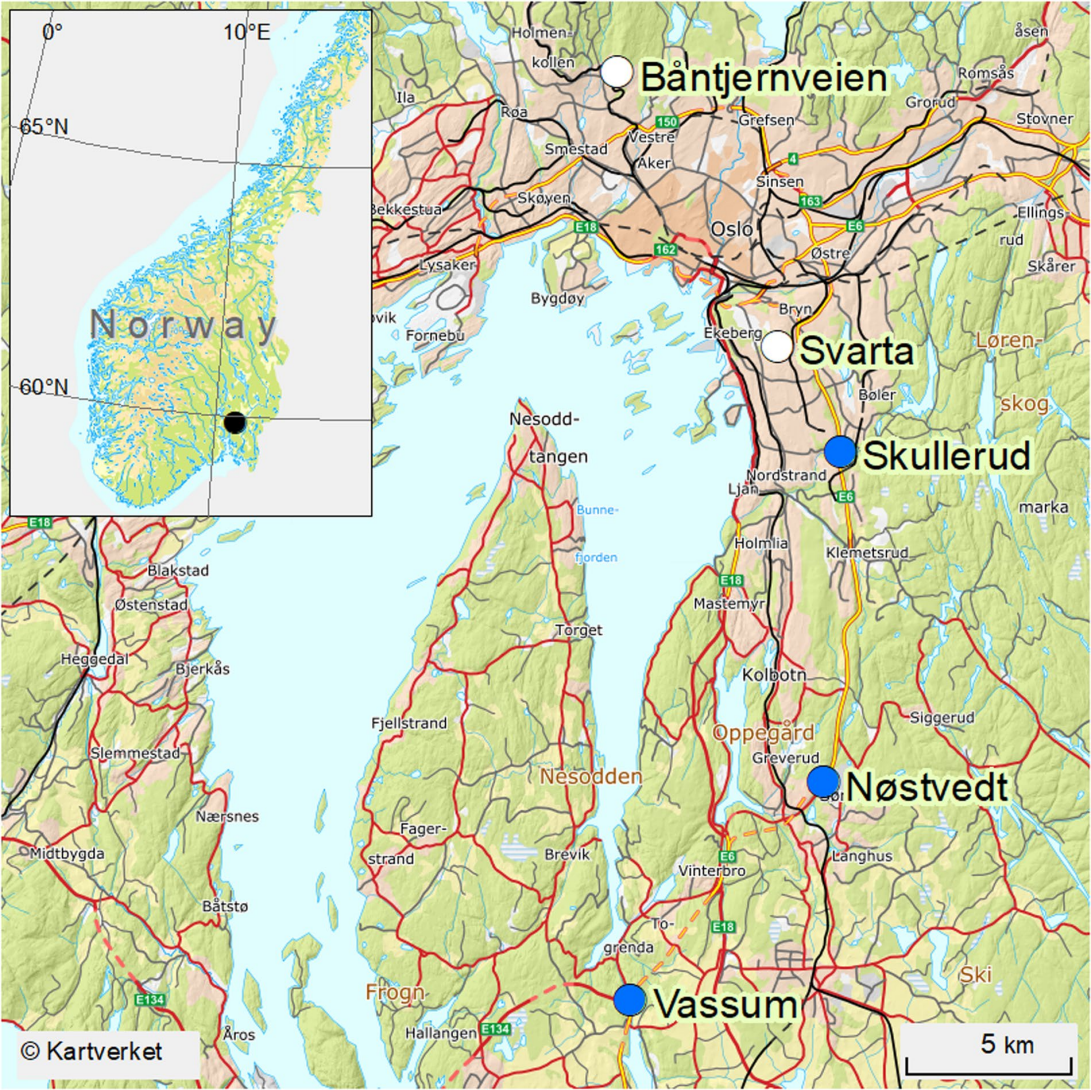
Map showing the various ponds included in the present study. Sedimentation ponds are marked with blue circles and natural ponds are marked with white circles. The map was created in Esri ArcGIS Desktop version 10.6.1.9270 (www.esri.com), using basemaps from the Norwegian Mapping Authority (© Kartverket, www.kartverket.no).

Three highway sedimentation ponds (Skullerud, Nøstvedt and Vassum) and two natural ponds (Svarta and Båntjernveien) were included in the study (Fig. 5 and Table 4). The ponds are situated in the greater Oslo area. Skullerud, Nøstvedt and Vassum receive runoff from the four-lane highway E6. In addition, the Vassum pond receives regularly tunnel wash water from three tunnels (Nordbytunnelen, Smiehagentunnelen and Vassumtunnelen). The three sedimentation ponds are slightly differently designed, but all have developed a dense vegetation within and along their edges. The natural pond Båntjernveien is in a dead-end residential street in the City of Oslo. The pond is surrounded by houses in one side and forest on the other side. Svarta is in a small forested area in the City of Oslo and its surroundings are often used for recreation purposes. Both ponds are far from the city center at the hillsides of the city and not influenced by traffic.

**Table 4:** Description of the ponds included in the present study.

Pond type	Sedimentation pond			Natural pond	
Pond name	Skullerud	Nøstvedt	Vassum	Svarta	Båntjernveien
**Coordinates (UTM33)**					
North Latitude	6643408.79	6633359.96	6626754.23	6646579.12	6654904.57
East Longitude	266614.62	266105.61	260253.21	264721.01	259847.98
Pond area (m^2^)^a^	Ca. 880	Ca. 260	Ca. 400	Ca. 1450	Ca. 820
Max. depth (m)	Ca. 1.5	<1	Ca. 1	Ca. 1.5	Ca. 4–6 m
Macrophyte cover (%)	Ca. 50	Ca. 80	Ca. 80	Ca. 30	Ca. 30
Constructed	1998/1999	2009	2000	—	—
Annual average daily traffic (AADT)^b^	70 100	46 915	66 326^c^	—	—
Runoff type	Highway runoff.	Highway runoff.	Highway and tunnel wash water runoff.	—	—
Pond type	Wet sedimentation pond; divided in a small closed pre-sedimentation basin and a lager main pond. Submerged inlet and outlet.	Wet sedimentation pond; divided in an open forebay made of concrete and a shallow main pond with several flow reducing weirs. Open inlet and submerged outlet.	Wet sedimentation pond; divided in an open forebay made of concrete and main pond. Submerged inlet and outlet.	Permanent water surface. No apparent inlet and outlet.	Permanent water surface. No apparent inlet and outlet.
Surrounding area	Surrounded by roads, partly situated below motorway bridge. Vegetation and a few trees surrounding the pond.	Situated beside a road tunnel. Surrounded by vegetation and trees/forest.	Surrounded by roads on all sides. Vegetation and a few trees surrounding the pond.	Surrounded by forest in a recreational area. Unimpacted from roads.	Dead-end residential area with houses on the west side and forest on east side of the pond. Little hard surface. Unimpacted from roads.

^a^Obtained from www.norgeskart.no (2019.03.22).

^b^Obtained from www.vegkart.no (2019.03.22).

^c^Based on the sum of AADT of the three tunnels Nordbytunnelen (E6), Smiehagentunnelen (E6) and Vassumtunnelen (E134).

### Sampling of sediment and chemical analysis

Sediment samples from the ponds were obtained from previous sampling campaigns in the period 2013 to 2018, while sediment from the two natural ponds were collected in 2017 and 2018. The sediment was collected by using a Van Veen grab, sampling approximately the upper 5 cm. Three to five subsamples from different places in the pond were combined to one composite sample. The sediment was stored on 100 mL incinerated glass jars and stored at −20 °C prior to analysis. The analysis of PAHs and alkylated PAHs were performed at NIVA research laboratory while metal analyses were performed by the accredited chemical laboratory Eurofins Norway (www.eurofins.no).

### PAH determination in sediment

#### Extraction of PAH

Approximately 2.5 g of freeze dried and homogenized sediment samples were weighed and extracted with 25 mL dichloromethane. During extraction the following internal standards (200 µL, 2000 ng/mL) were added: naphthalene-*d8*, biphenyl-*d10*, acenaphthylene-*d8*, dibenzothiophene-*d10*, pyrene-*d10*, benzo(*a*)anthracene-*d12* and perylene-*d12*). The extracts were thereafter placed in an ultrasonic bath (1 h) and centrifuged for 5 minutes at 3000 RPM. The extraction process was repeated once, and the aliquots combined. The extracts were then concentrated, added ethyl acetate (LS-MS grade) and cleaned by transfer to Eppendorf tubes with filters (0.2 µM nylon filters, Costar, Spin-X) and centrifuged (13 000 RPM, 1 min.). The extract was further cleaned by Gel Permeation Chromatography (GPC). A small amount of cyclohexane was added, and the extracts were concentrated by a gentle stream of nitrogen and analysed by means of GC/MS.

#### GC/MS-analyses

Analyses were performed on an Agilent 6890 N gas chromatograph (GC) linked to an Agilent 5973 N mass selective detector (MSD) operated in single ion monitoring mode (SIM). The ionisation was electron impact (70 eV). Separation of individual PAHs was done on a DB5 column (30 m, 0.25 mm inner diameter and 0.25 µm film thickness, Agilent JW Scientific). The injection was pulsed splitless injection (2 µL injection, pulse pressure 20 psi for 1.2 minutes, injection temperature 300 °C). The carrier gas was He (1.2 mL/min). The GC oven temperature program started at 60 °C for 2 min. and was raised first to 250 °C (7 °C/min) and thereafter 310 °C (15 °C/min) and kept at 310 °C for 6 min. Temperatures for the ion source and quadrupole and transfer line were 230, 150 °C and 300 °C respectively. A total of 33 mass-to-charge ratios (m/z) divided into 6 groups were acquired in SIM mode (Table 5). The dwell times for each *m/z* varied between 30 to 45 ms within groups.

**Table 5:** List of compounds, m/z and corresponding groups of GC/MS-SIM.

Compound	*m*/*z*	Group	Compound	*m*/*z*	Group
Naphthalene	128	1	C3-Fluorenes*	208	3
Naphthalene-*d8*	136	1	C2-Dibenzothiophenes	212	4
C1-Naphthalenes	142	1	Pyrene-*d10*	212	4
Acenaphthylene	152	2	C1-Pyrene/Fluoranthene*	216	5
Acenaphthene	154	2	C3-Phenanthrenes	220	5
C2-Naphthalenes	156	2	C3-Dibenzothiophenes	226	4
Acenaphthylen-*d8*	160	2	Benzo(a)anthracene	228	5
Biphenyl-*d10*	164	2	Chrysen	228	5
Fluoren	166	3	C2-Pyrene/Fluoranthene*	230	5
C3-Naphthalene	170	2	C4-Phenanthrenes*	234	5
Phenanthrene	178	3	Benzo(a)anthracene-*d12*	240	5
Anthracene	178	3	C1-Chrysenes*	242	5
C1-Fluorenes*	180	3	Benzo(b,j)fluoranthene	252	6
C4-Naphthalenes*	184	3	Benzo(k)fluoranthene	252	6
Dibenzothiophene	184	3	Benzo(e)pyrene	252	6
Dibenzothiophene-*d10*	192	3	Benzo(a)pyrene	252	6
C1-Phenanthrenes	192	4	Perylene	252	6
C2-Fluorenes*	194	3	C2-Chrysenes*	256	6
C1-Dibenzothiophenes	198	4	Perylene-*d12*	264	6
Fluoranthene	202	4	Indeno(1,2,3-cd)pyrene	276	6
Pyren	202	5	Benzo(ghi)perylene	276	6
C2-Phenanthrenes	206	4	Dibenz(ac/ah)anthracene	278	6

Internal standards are denoted with d8, d10 and d12. *These compounds were not analysed for all samples in this study, and consequently are not reported for any samples.

#### Quality assurance

A certified reference material, SRM-1944 (NIST), was analysed together with the samples. The median of deviations between measured concentrations and certified values for all PAHs of interest was 15% (range of −3 to −37%). Calibration was performed based on 7 standards in the range of 1.5–4000 ng/mL. In addition to the PAH16 compounds, the following standards were included: benzo(e)pyrene, perylene, 2-methyl naphthalene; 2,6-dimethyl naphthalene; 2-isopropyl naphthalene; 1,4,6,7-tetramethyl naphthalene; 9-mehtyl phenanthrene; 9-ethyl phenanthrene; 1,2,6-trimethyl phenanthrene; 1,2,6,9-tetramethyl phenanthrene; 4-methyl dibenzothiophene; 4-ethyl dibenzothiophene; 4-propyl dibenzothiophene; 1-methyl fluorene; 1,7-dimethyl fluorene; 9-n-propyl fluorene; 1-methyl pyrene; 4,5-dimethyl pyrene; 4-methyl chrysene and 6-ethyl chrysene. Quantification of alkylated PAHs was done under the assumption of equal response as the alkylated standards that formed the calibration curves.

### Sampling of dragonflies

In total 60 dragonfly nymphs from the genus *Aeshna* were collected between 29 and 30 of August 2018. Several rain events had occurred during a two-week period prior to sampling. The nymphs were collected along the riparian vegetation around the ponds by using a net. Twelve nymphs from each pond with approximately the same size were handpicked and placed separately in 50 mL Falcon tubes to avoid cannibalism. The Falcon tubes with nymphs were stored in the dark and in the cold in boxes with ice during the transportation back to the NIVA research laboratory. The transportation time from the ponds to the laboratory was less than one hour. Processing of the nymphs for the comet assay started immediately after arrival to the laboratory.

The nymphs were determined to species level (after the comet assay) by using the taxonomic keys in Sahlén^57^ and Nilsson^58^.

### Comet assay

After protocol optimization (see supplementary information for details on method optimization steps), 12 nymphs from each sedimentation and natural ponds were processed for the comet assay. Upon arrival, falcon tubes were placed in racks and lids were opened to allow gas exchange. Individual nymphs were placed on ice for a few minutes, after which their length was measured with a caliper before one of the legs was dissected off. Hemolymph was extracted using microcapillary tubes and immediately transferred into a 0.2 mL PCR tube containing 20 µL PBS buffer (without Ca^2+^/Mg^2+^, pH 7.4) with PTU (phenylthiourea) 0.07% (PBS-PTU) to avoid coagulation of the cells. Up to 20 µL hemolymph was extracted from each individual and no pooling of samples was needed. After extraction, haemolymph’s cell suspension was adjusted to 2.2 × 10^5^ cells/mL with PBS-PTU and cell viability checked using the trypan blue exclusion assay by randomly counting 50 cells for each biological replicate. To increase the number of samples that could be handled simultaneously, the high throughput version of the comet assay with 12 mini gels on one microscope slide coated with polyester film substrate (GelBond®films) was used^59^. Cells were resuspended in 1% low melting point agarose at 37 °C and placed on a cold microscope slide coated with GelBond®films in 7 µL aliquots. Lysis was performed overnight in lysis buffer (2.5 M NaCl, 0.1 M Na_2_EDTA, 0.01 M Tris, 0.2 M NaOH, 1% Triton X-100, pH 10) at 4 °C. Slides were prepared in duplicate according to three different treatments: 1) Lysis only (to measure DNA strand breaks and alkali-labile sites); 2) Incubation with FPG buffer after lysis; and 3) Incubation with FPG after lysis (to measure oxidised guanine, oxidised bases). After lysis, slides from treatment 2 and 3 were rinsed 3 × 5 min in enzyme incubation buffer (40 mM HEPES, 0.1 M KCl, 0.5 mM EDTA, 0.2 mg/mL bovine serum albumin, pH adjusted to 8.0) at room temperature. Slides were immersed in either (prewarmed) enzyme buffer or FPG (prepared as a crude extract from an over-producing strain of *Escherichia coli*) diluted 1:4500 in the buffer. Slides were incubated for 30 min at 37 °C. For unwinding, slides from all 3 treatments were immersed in cold electrophoresis solution (0.3 M NaOH, 0.001 M Na_2_EDTA, pH > 13) for 40 min. Electrophoresis was carried out in cold, fresh electrophoresis solution for 20 min at 8 °C, 25 V giving 0.8 V/cm across the platform, with circulation of electrophoresis solution. After electrophoresis, slides were neutralized with neutralisation buffer (PBS), washed with H_2_O, 70% ethanol (5 min) and absolute ethanol (5 min), and left to dry at room temperature. Slides were stained with SYBR®Gold Nucleic Acid Gel Stain (Life Technologies, Paisley, UK) and fifty randomly chosen cells per replicate were scored using the Comet IV analysis software (Perceptive Instruments Ltd., Bury St. Edmunds, UK). Tail intensity (% Tail DNA), defined as the percentage of DNA migrated from the head of the comet into the tail was used as a measure of DNA, as it has been shown to be the most meaningful endpoint to assess genotoxicity^60^. The mean percentage (%) of DNA in the tail per biological replicate was calculated using the median values of % tail DNA from the 50 comets from each replicate. Net FPG-sensitive sites were calculated as the difference between the scoring results obtained for treatment 2 (incubation with FPG buffer after lysis) and treatment 3 (incubation with FPG after lysis).

### Statistics

Data treatment, statistical analyses and graphical outputs were performed with JMP 14.0.0. (SAS Institute Inc), Canoco 5.11. (Microcomputer Power), R version 3.6.0 (the R Core Team, 2019) and RStudio version 1.2.1335 (RStudio, Inc). Canoco was used for the regression models while R and RStudio (package “ape” version 5.3) were used to evaluate any spatial autocorrelation in the residuals of the regression models.

Univariate statistics were used to disclose any statistically significant differences in results obtained from the comet assay. Whenever assumption of normality and equal variance on either original or log-transformed data were met, parametric t-test and one-way ANOVA followed by a Tukey post hoc test were performed. Level of statistical significance was set to *p* < 0.05. As there were no statistical difference in comet results between the two abundant species (*A*. *juncea* and *A*. *cyanea*) neither when pooled across ponds nor in the separate ponds Svarta and Nøstvedt, we choose to pool all the species into *Aeshna sp*.

Permutation based linear and multiple regressions using the multivariate statistical method Redundancy Analysis (RDA) were used to assess the relationship between the comet results and pollution levels in the ponds, as well as the relationship between size of the nymphs and pollution levels. The method is described in detail in Šmilauer and Lepš^61^. In RDA the significance of the explanatory variables is tested by using Monte Carlo permutation tests where the distribution of the original data is simulated by permutations deriving the pseudo F ratio which is the counterpart of the F ratio in parametric significance test of a regression model. We used 1999 permutations for our tests, and the level of significance was set to *p* < 0.05. By using a permutation-based model, the assumptions of normality required in parametric regression tests are abandoned.

We utilized Moran’s Index (I) to reveal any spatial autocorrelation in the residuals of the regression models. Moran’s I is a correlation coefficient ranging from −1 (perfect dispersion) to 1 (perfect clustering) that measures the overall spatial autocorrelation in the data. The null hypothesis was that there is no spatial clustering between the residuals and the geographic positions of the ponds in the study area.

## Supplementary information

Supplementary Information: Road related pollutants induced DNA damage in dragonfly (Odonata, Anisoptera) nymphs living in highway sedimentation ponds

## Data Availability

The datasets generated during and/or analysed during the current study are available from the corresponding author on reasonable request.

## References

[1] European Commission. Road Transport - A change of gear. 16 (EC, Luxembourg, 2012).

[2] European Environment Agency. Monitoring progress of Europe’s transport sector towards its environment, health and climate objectives. 11 (EEA, Online, 2017).

[3] Huber M, Welker A, Helmreich B (2016). Critical review of heavy metal pollution of traffic area runoff: Occurrence, influencing factors, and partitioning. Science of The Total Environment.

[4] Brown JN, Peake BM (2006). Sources of heavy metals and polycyclic aromatic hydrocarbons in urban stormwater runoff. Science of The Total Environment.

[5] Hwang HM, Fiala MJ, Park D, Wade TL (2016). Review of pollutants in urban road dust and stormwater runoff: part 1. Heavy metals released from vehicles. International Journal of Urban Sciences.

[6] Gobel P, Dierkes C, Coldewey WC (2007). Storm water runoff concentration matrix for urban areas. Journal of Contaminant Hydrology.

[7] Maltby L, Forrow DM, Boxall ABA, Calow P, Betton CI (1995). The effects of motorway runoff on fresh-water ecosystems. 1. field-study. Environmental Toxicology and Chemistry.

[8] Meland S (2010). Chemical and ecological effects of contaminated tunnel wash water runoff to a small Norwegian stream. Science of The Total Environment.

[9] Meland, S. Management of contaminated runoff water. Current practice and Future Research Needs. 84 (Conference of European Directors of Roads (CEDR), Brussels, 2016).

[10] Hvitved-Jacobsen, T., Vollertsen, J. & Haaning Nielsen, A. *Urban and Highway Stormwater Pollution*. *Concepts and Engineering*. (CRC Press, 2010).

[11] Istenic D, Arias CA, Matamoros V, Vollertsen J, Brix H (2011). Elimination and accumulation of polycyclic aromatic hydrocarbons in urban stormwater wet detention ponds. Water Science and Technology.

[12] Herrmann J (2012). Chemical and biological benefits in a stormwater wetland in Kalmar, SE Sweden. Limnologica.

[13] Briers RA (2014). Invertebrate Communities and Environmental Conditions in a Series of Urban Drainage Ponds in Eastern Scotland: Implications for Biodiversity and Conservation Value of SUDS. Clean-Soil Air Water.

[14] Le Viol I, Chiron F, Julliard R, Kerbiriou C (2012). More amphibians than expected in highway stormwater ponds. Ecological Engineering.

[15] Le Viol I, Mocq J, Julliard R, Kerbiriou C (2009). The contribution of motorway stormwater retention ponds to the biodiversity of aquatic macroinvertebrates. Biol. Conserv..

[16] Sun Z (2018). Aquatic biodiversity in sedimentation ponds receiving road runoff – What are the key drivers?. Science of The Total Environment.

[17] Hill MJ (2017). Urban ponds as an aquatic biodiversity resource in modified landscapes. Global Change Biology.

[18] Sun Z (2019). Impact of environmental factors on aquatic biodiversity in roadside stormwater ponds. Scientific Reports.

[19] Holtmann L, Juchem M, Brüggeshemke J, Möhlmeyer A, Fartmann T (2018). Stormwater ponds promote dragonfly (Odonata) species richness and density in urban areas. Ecological Engineering.

[20] Villalobos-Jimenez G, Dunn AM, Hassall C (2016). Dragonflies and damselflies (Odonata) in urban ecosystems: A review. Eur. J. Entomol..

[21] Brand AB, Snodgrass JW, Gallagher MT, Casey RE, Van Meter R (2010). Lethal and Sublethal Effects of Embryonic and Larval Exposure of Hyla versicolor to Stormwater Pond Sediments. Archives of Environmental Contamination and Toxicology.

[22] Snodgrass JW, Casey RE, Joseph D, Simon JA (2008). Microcosm investigations of stormwater pond sediment toxicity to embryonic and larval amphibians: Variation in sensitivity among species. Environmental Pollution.

[23] Grung Merete, Petersen Karina, Fjeld Eirik, Allan Ian, Christensen Jan H., Malmqvist Linus M.V., Meland Sondre, Ranneklev Sissel (2016). PAH related effects on fish in sedimentation ponds for road runoff and potential transfer of PAHs from sediment to biota. Science of The Total Environment.

[24] Meland, S., Damsgård, M. B., Skipperud, L. & Heier, L. S. In *Urban Environment* (eds Sébastien Rauch, Gregory Morrison, Stefan Norra, & Nina Schleicher) Ch. 44, 495–505 (Springer Netherlands, 2013).

[25] Johansen, S. L. *Element accumulation and levels of four biomarkers in common frog* (*Rana temporaria*) *tadpoles in two sedimentation ponds and naturally occurring pond* MSc-thesis thesis, Norwegian University of Life Sciences, (2013).

[26] Haile TM (2016). Cytotoxic and genotoxic activities of waters and sediments from highway and parking lot runoffs. Water Science and Technology.

[27] Singh NP, McCoy MT, Tice RR, Schneider EL (1988). A simple technique for quantitation of low levels of DNA damage in individual cells. Exp Cell Res.

[28] Azqueta, A. & Collins, A. R. In *Genotoxicity and* DNA *Repair:* A *Practical Approach Methods in Pharmacology and Toxicology* (eds L. María Sierra & Isabel Gaivão) Ch. 12, 483 (Humana Press, 2014).

[29] Matzenbacher CA (2017). DNA damage induced by coal dust, fly and bottom ash from coal combustion evaluated using the micronucleus test and comet assay *in vitro*. Journal of Hazardous Materials.

[30] Augustyniak M, Gladysz M, Dziewięcka M (2016). The Comet assay in insects—Status, prospects and benefits for science. Mutation Research/Reviews in Mutation Research.

[31] Lund Johansen S, Thygesen H, Meland S (2014). Kjemisk karakterisering av sediment i rensebassenger for vegavrenning (In Norwegian). Vann.

[32] Bartlett AJ, Rochfort Q, Brown LR, Marsalek J (2012). Causes of toxicity to Hyalella azteca in a stormwater management facility receiving highway runoff and snowmelt. Part I: Polycyclic aromatic hydrocarbons and metals. Science of The Total Environment.

[33] Wayland M, Headley JV, Peru KM, Crosley R, Brownlee BG (2008). Levels of polycyclic aromatic hydrocarbons and dibenzothiophenes in wetland sediments and aquatic insects in the oil sands area of Northeastern Alberta, Canada. Environmental Monitoring and Assessment.

[34] Richter-Brockmann S, Achten C (2018). Analysis and toxicity of 59 PAH in petrogenic and pyrogenic environmental samples including dibenzopyrenes, 7H-benzo c fluorene, 5-methylchrysene and 1-methylpyrene. Chemosphere.

[35] Starzec P, Lind BOB, Lanngren A, Lindgren A, Svenson T (2005). Technical and environmental functioning of detention ponds for the treatment of highway and road runoff. Water Air and Soil Pollution.

[36] de Lapuente, J. *et al*. The Comet Assay and its applications in the field of ecotoxicology: a mature tool that continues to expand its perspectives. *Frontiers in Genetics* **6**, 10.3389/fgene.2015.00180 (2015). 10.3389/fgene.2015.00180 PMC4454841 26089833

[37] Gajski G (2019). The comet assay in animal models: From bugs to whales – (Part 1 Invertebrates). Mutation Research/Reviews in Mutation Research.

[38] Ball A, Truskewycz A (2013). Polyaromatic hydrocarbon exposure: an ecological impact ambiguity. Environmental Science and Pollution Research.

[39] Ewa B, Danuta M-Š (2017). Polycyclic aromatic hydrocarbons and PAH-related DNA adducts. Journal of applied genetics.

[40] Vicentini M (2017). Benzo(a) pyrene Exposure Causes Genotoxic and Biochemical Changes in the Midge Larvae of Chironomus sancticaroli Strixino & Strixino (Diptera: Chironomidae). Neotropical Entomology.

[41] Morais GDS, Pesenti EC, Cestari MM, Navarro-Silva MA (2014). Genotoxic effect of Phenanthrene on Chironomus sancticaroli (Diptera: Chironomidae). Zoologia (Curitiba).

[42] Richardi VS (2018). Effects of phenanthrene on different levels of biological organization in larvae of the sediment-dwelling invertebrate Chironomus sancticaroli (Diptera: Chironomidae). Environmental Pollution.

[43] Wu XY (2016). A review of toxicity and mechanisms of individual and mixtures of heavy metals in the environment. Environmental Science and Pollution Research.

[44] Martinez-Alvarez RM, Morales AE, Sanz A (2005). Antioxidant defenses in fish: Biotic and abiotic factors. Reviews in Fish Biology and Fisheries.

[45] Lushchak VI (2011). Environmentally induced oxidative stress in aquatic animals. Aquatic Toxicology.

[46] Hare L (1992). Aquatic insects and trace metals - Bioavailability, bioaccumulation and toxicity. Critical Reviews in Toxicology.

[47] Fleeger JW, Gust KA, Marlborough SJ, Tita G (2007). Mixtures of metals and polynuclear aromatic hydrocarbons elicit complex, nonadditive toxicological interactions in meiobenthic copepods. Environmental Toxicology and Chemistry.

[48] Stephansen, D. A. *et al*. In *Urban Environment* (eds Sébastien Rauch, Gregory Morrison, Stefan Norra, & Nina Schleicher) Ch. 43, 485-494 (Springer Netherlands, 2013).

[49] Azqueta A, Collins AR (2013). The essential comet assay: a comprehensive guide to measuring DNA damage and repair. Archives of Toxicology.

[50] Azqueta A, Arbillaga L, Lopez de Cerain A, Collins A (2013). Enhancing the sensitivity of the comet assay as a genotoxicity test, by combining it with bacterial repair enzyme FPG. Mutagenesis.

[51] Mitchelmore CL, Chipman JK (1998). DNA strand breakage in aquatic organisms and the potential value of the comet assay in environmental monitoring. Mutation research.

[52] Nummelin M, Lodenius M, Tulisalo E, Hirvonen H, Alanko T (2007). Predatory insects as bioindicators of heavy metal pollution. Environmental Pollution.

[53] Popova ON, Haritonov AY, Anishchenko OV, Gladyshev MI (2016). Export of biomass and metals from aquatic to terrestrial ecosystems via the emergence of dragonflies (Insecta: Odonata). Contemporary Problems of Ecology.

[54] Tüzün N, Debecker S, Op de Beeck L, Stoks R (2015). Urbanisation shapes behavioural responses to a pesticide. Aquatic Toxicology.

[55] Bartlett AJ, Rochfort Q, Brown LR, Marsalek J (2012). Causes of toxicity to Hyalella azteca in a stormwater management facility receiving highway runoff and snowmelt. Part II: Salts, nutrients, and water quality. Science of The Total Environment.

[56] Statens vegvesen. Håndbok N500 Vegtunneler (In Norwegian). 88 (Statens vegvesen, Oslo, 2016).

[57] Sahlén, G. *Sveriges trollsländor* (In Swedish). 162 (Fältbiologerna, 1996).

[58] Nilsson, A. *Aquatic insects of North Europe: a taxonomic handbook: Vol*. *2: Odonata - Diptera*. (Apollo Books, 1997).

[59] Shaposhnikov S (2010). Twelve-gel slide format optimised for comet assay and fluorescent *in situ* hybridisation. Toxicol Lett.

[60] Kumaravel TS, Jha AN (2006). Reliable Comet assay measurements for detecting DNA damage induced by ionising radiation and chemicals. Mutation research.

[61] Šmilauer, P. & Lepš, J. *Multivariate analysis of ecological data using CANOCO 5*. 2nd ed. edn, (Cambridge University Press, 2014).

[62] Direktoratsgruppen vanndirektivet. Klassifisering av miljøtilstand i vann. Økologisk og kjemisk klassifiseringssytem for kystvann, grunnvann, innsjøer og elver. Veileder 02:2018 (In Norwegian). 222 (www.vannportalen.no, 2018).

[63] Srogi K (2007). Monitoring of environmental exposure to polycyclic aromatic hydrocarbons: a review. Environmental Chemistry Letters.

