# Supplementary material for: Road related pollutants induced DNA damage in dragonfly nymphs (Odonata, Anisoptera) living in highway sedimentation ponds
Source: Sci Rep. 2019 Nov 5;9:16002. doi: 10.1038/s41598-019-52207-4 (PMC6831790; doi:10.1038/s41598-019-52207-4)
Supplement: Supplementary file 1 — Supplementary Information: Road related pollutants induced DNA damage in dragonfly (Odonata, Anisoptera) nymphs living in highway sedimentation ponds [file 41598_2019_52207_MOESM1_ESM.pdf]

Supplementary Information

Road related pollutants induced DNA damage in dragonfly nymphs (Odonata, Anisoptera) living in highway sedimentation ponds

Sondre Meland[a,b,*], Tânia Gomes[a], Karina Petersen[a], Johnny Håll[a], Espen Lund[a], Alfhild Kringstad[a], Merete Grung[a]

[a]Norwegian Institute for Water Research (NIVA), Gaustadalléen 21, 0349 Oslo, Norway

[b]Norwegian University for Life Sciences (NMBU), Faculty of Environmental Sciences and Natural Resource Management, PO 5003, 1432 Ås, Norway

*Corresponding author: Sondre Meland, Norwegian Institute for Water Research (NIVA), Gaustadalléen 21, 0349 Oslo, Norway. E-mail address sondre.meland@niva.no, phone +47 970 37 586

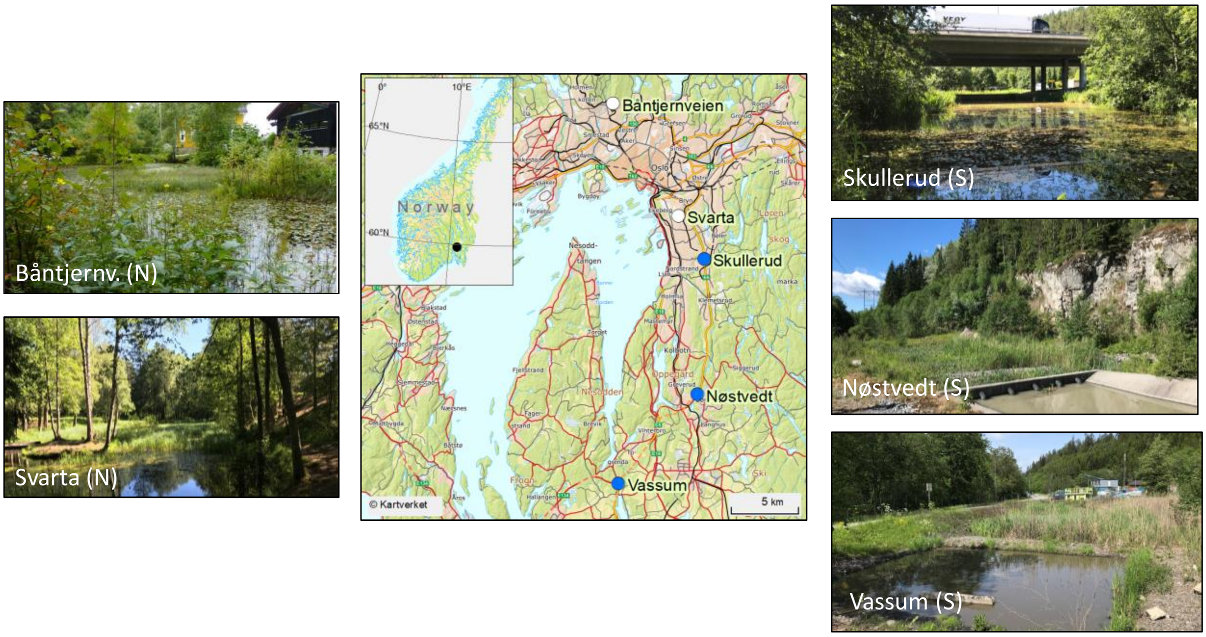

Supplementary photos of the ponds and their location displayed in a map. Natural ponds are indicated with (N) and Sedimentation ponds are indicated with (S). All photos: Sondre Meland. The map was created in Esri ArcGIS Desktop version 10.6.1.9270 (www.esri.com), using basemaps from the Norwegian Mapping Authority (© Kartverket, www.kartverket.no).

Supplementary Table S 1. Data on the dragonfly nymphs from the various ponds (N = natural pond, S = sedimentation pond), including output from the comet assay and cell viability. Number of nymphs (n) is 60.

Pond	Ind no.	Species	Size (cm)	Cell viability (%)	% Tail DNA (net FPG)	% Tail DNA (LYS)	% Tail DNA (LYS + net FPG)
Vassum (S)	4	Aeshna cyanea	3.5	90	18.7	19.3	38.0
Vassum (S)	7	Aeshna cyanea	3.0	90	13.8	3.9	17.7
Vassum (S)	3	Aeshna cyanea	3.1	100	21.9	5.7	27.6
Vassum (S)	12	Aeshna cyanea	2.4	96	23.2	17.4	40.6
Vassum (S)	16	Aeshna cyanea	2.1	98	9.1	35.9	45.0
Vassum (S)	8	Aeshna cyanea	2.3	86	-	-	-
Vassum (S)	1	Aeshna cyanea	2.8	96	16.2	13.6	29.7
Vassum (S)	6	Aeshna cyanea	2.4	98	13.1	5.0	18.1
Vassum (S)	10	Aeshna cyanea	2.6	80	33.1	1.4	34.5
Vassum (S)	2	Aeshna cyanea	2.9	100	17.6	1.1	18.7
Vassum (S)	13	Aeshna cyanea	2.4	98	27.7	1.8	29.6
Vassum (S)	11	Aeshna cyanea	2.2	94	26.3	2.3	28.6
Svarta (N)	8	Aeshna cyanea	3.4	98	18.3	1.4	19.6
Svarta (N)	1	Aeshna juncea	3.8	96	30.7	0.4	31.1
Svarta (N)	7	Aeshna cyanea	3.2	96	20.0	0.4	20.4
Svarta (N)	5	Aeshna cyanea	2.9	94	36.8	1.3	38.1
Svarta (N)	12	Aeshna cyanea	2.5	92	29.0	1.0	30.0
Svarta (N)	4	Aeshna juncea	3.0	100	21.3	5.1	26.4

Pond	Ind no.	Species	Size (cm)	Cell viability (%)	% Tail DNA (net FPG)	% Tail DNA (LYS)	% Tail DNA (LYS + net FPG)
Svarta (N)	6	Aeshna cyanea	3.7	94	27.3	0.5	27.8
Svarta (N)	2	Aeshna juncea	3.7	100	21.3	0.7	22.0
Svarta (N)	3	Aeshna juncea	3.4	100	20.6	1.5	22.1
Svarta (N)	11	Aeshna juncea	3.1	96	30.4	2.4	32.8
Svarta (N)	9	Aeshna cyanea	3.5	94	24.7	0.5	25.2
Svarta (N)	14	Aeshna juncea	2.4	100	23.1	4.8	27.9
Skullerud (S)	3	Aeshna cyanea	4.0	96	21.9	16.8	38.7
Skullerud (S)	9	Aeshna cyanea	2.9	84	26.9	10.6	37.6
Skullerud (S)	1	Aeshna juncea	3.7	90	27.8	4.8	32.6
Skullerud (S)	7	Aeshna cyanea	3.0	96	26.5	2.2	28.6
Skullerud (S)	14	Aeshna cyanea	2.5	100	18.9	16.3	35.2
Skullerud (S)	8	Aeshna cyanea	3.1	84	3.0	14.3	17.3
Skullerud (S)	2	Aeshna cyanea	3.5	90	25.1	20.0	45.1
Skullerud (S)	12	Aeshna grandis	3.2	92	11.9	0.8	12.7
Skullerud (S)	4	Aeshna cyanea	3.4	86	25.4	2.7	28.0
Skullerud (S)	10	Aeshna cyanea	2.9	100	23.7	1.7	25.4
Skullerud (S)	11	Aeshna cyanea	2.4	94	22.0	2.4	24.3
Skullerud (S)	6	Aeshna cyanea	3.0	100	27.5	3.7	31.2
Nøstvedt (S)	5	Aeshna cyanea	3.2	96	35.2	10.2	45.3
Nøstvedt (S)	6	Aeshna juncea	2.9	98	25.7	6.9	32.5
Nøstvedt (S)	9	Aeshna juncea	2.9	92	24.4	10.5	34.9
Nøstvedt (S)	14	Aeshna cyanea	2.6	100	17.9	10.9	28.9
Nøstvedt (S)	15	Aeshna cyanea	2.5	100	15.4	8.3	23.7
Nøstvedt (S)	3	Aeshna cyanea	3.3	96	13.1	3.4	16.4
Nøstvedt (S)	7	Aeshna cyanea	3.2	98	11.1	4.8	15.9
Nøstvedt (S)	10	Aeshna juncea	2.8	94	12.5	2.3	14.8
Nøstvedt (S)	13	Aeshna juncea	2.9	84	35.4	0.6	36.1
Nøstvedt (S)	12	Aeshna sp.	3.1	94	25.5	0.4	25.9
Nøstvedt (S)	8	Aeshna grandis	2.8	96	27.7	4.7	32.4
Nøstvedt (S)	11	Aeshna cyanea	3.1	96	20.3	2.8	23.1
Båntjernv (N)	1	Aeshna cyanea	3.7	100	27.4	0.67	28.1
Båntjernv (N)	3	Aeshna juncea	3.3	100	18.8	0.4	19.2
Båntjernv (N)	7	Aeshna cyanea	3.8	90	18.6	4.6	23.2
Båntjernv (N)	8	Aeshna cyanea	3.1	100	25.9	1.3	27.1
Båntjernv (N)	2	Aeshna juncea	3.9	98	32.3	0.7	33.0
Båntjernv (N)	4	Aeshna juncea	3.1	98	27.6	1.6	29.2
Båntjernv (N)	13	Aeshna cyanea	2.4	96	11.1	12.6	23.7
Båntjernv (N)	6	Aeshna cyanea	3.4	92	23.9	2.0	25.9
Båntjernv (N)	11	Aeshna cyanea	2.6	98	23.6	0.3	23.9
Båntjernv (N)	10	Aeshna cyanea	3.2	98	22.6	0.4	22.9
Båntjernv (N)	12	Aeshna cyanea	2.0	98	15.1	10.3	25.4
Båntjernv (N)	9	Aeshna cyanea	3.5	100	33.4	1.3	34.7

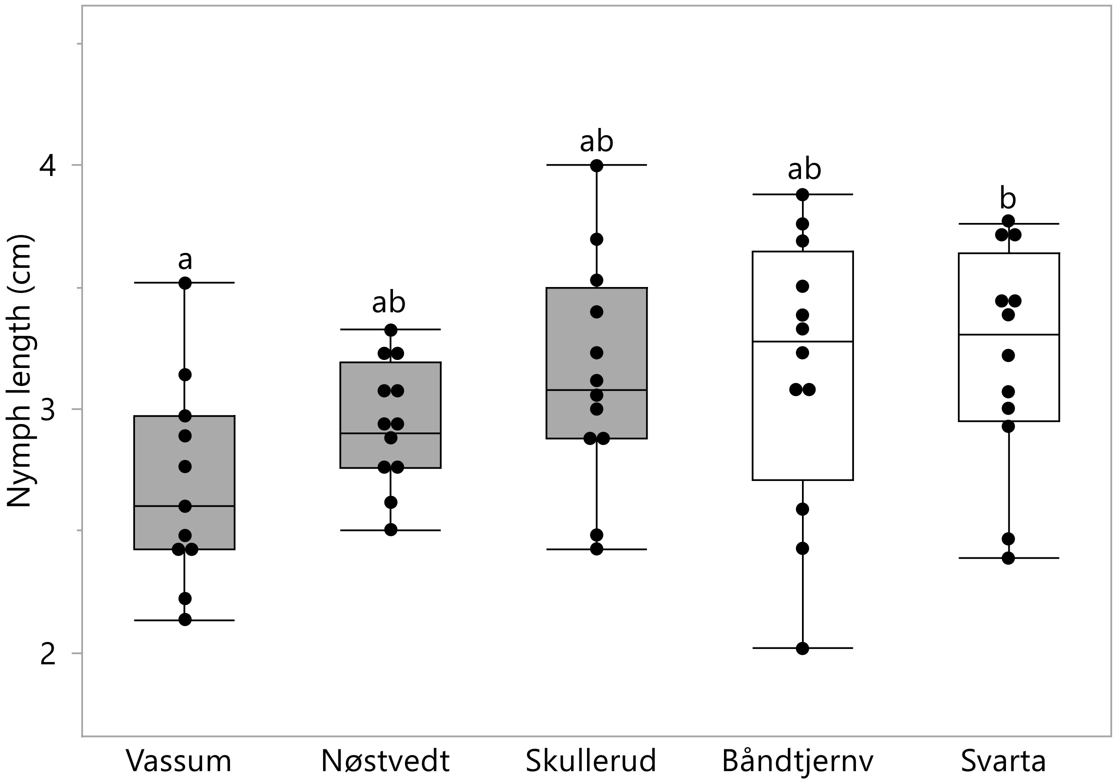

Supplementary Figure S 1. Box-plot showing the length of the sampled *Aeshna sp*. nymphs living in highway sedimentation ponds (grey) and natural ponds (white). Individual measurements are included as black points (n = 11 – 12). Box-plots with different letters indicate statistically significant differences in length of nymphs from different ponds. ANOVA (p = 0.039, n = 59).

Supplementary Table S 2. Descriptive statistics of DNA damage in dragonfly nymphs from the various ponds (N = natural, S = sedimentation pond) obtained from the comet assay.

	% Tail DNA (LYS)					% Tail DNA (net FPG)				% Tail DNA (LYS + net FPG)			
Pond	N	Mean	Median	Min	Max	Mean	Median	Min	Max	Mean	Median	Min	Max
Vassum (S)	11	9.8	5	1.1	35.9	20.1	18.7	9.1	33.1	29.8	29.6	17.7	45
Skullerud (S)	12	8	4.3	0.8	20	21.7	24.4	3	27.8	29.7	29.9	12.7	45.1
Nøstvedt (S)	12	5.5	4.8	0.4	10.9	22	22.4	11.1	35.4	27.5	27.4	14.8	45.3
Båntjernv (N)	12	3	1.3	0.3	12.6	23.4	23.8	11.1	33.4	26.4	25.7	19.2	34.7
Svarta (N)	12	1.7	1.2	0.4	5.1	25.3	23.9	18.3	36.8	27	27.1	19.6	38.1
All	59	5.5	2.4	0.3	35.9	22.5	23.2	3	36.8	28	27.9	12.7	45.3

Supplementary Table S 3. Output from the permutation based linear regression (a-d) and multiple regression (e-h) analyses between mean DNA damage and mean nymph length (dependent variables) and sediment pollutants (independent variables). The statistical tests are derived from 1999 permutations using Monte Carlo permutation test. Simple term effects (a-d) show the individual effect of each variable alone, and conditional term effects (e-h) show the increased effect of a variable considering already selected variables. Significant variables are highlighted in grey. The variables are sorted in descending order of their explained variation. * indicates a negative relationship between the pollutants and the DNA damage. N = 5.

**a) Linear regression - % Tail DNA (LYS)**			
**Simple Term Effects:**			
**Name**	**Explains %**	**pseudo-F**	**P**
Tot PAH+$PAH_{alkylated}$	96.6	86.1	0.007
Zinc	90.6	28.9	0.0215
Copper	87.3	20.7	0.051
Nickel	62.8	5.1	0.075
Lead	47.6	2.7	0.183
Cadmium	13.9	0.5	0.7055

**e) Multiple linear regression - % Tail DNA (LYS)**			
**Conditional Term Effects:**			
**Name**	**Explains %**	**pseudo-F**	**P**
Tot PAH+$PAH_{alkylated}$	96.6	86.1	0.0065
Lead	1.8	2.3	0.244
Nickel	1.4	11.3	0.1295
Zinc	0.1	<0.1	1.

**b) Linear regression - % Tail DNA (net FPG)***			
**Simple Term Effects:**			
**Name**	**Explains %**	**pseudo-F**	**P**
Zinc	93.4	42.1	0.0125
Tot PAH+$PAH_{alkylated}$	79.9	12.0	0.0275
Copper	71.7	7.6	0.065
Nickel	70.8	7.3	0.0975
Lead	33.9	1.5	0.2885
Cadmium	3.9	0.1	0.7115

**f) Multiple linear regression - % Tail DNA (net FPG)***			
**Conditional Term Effects:**			
**Name**	**Explains %**	**pseudo-F**	**P**
Zinc	93.4	42.1	0.0145
Cadmium	1.6	0.7	0.5655
Tot PAH+$PAH_{alkylated}$	4.8	23.2	0.125
Lead	0.2	<0.1	1.

**c) Linear regression - % Tail DNA (LYS + net FPG)**			
**Simple Term Effects:**			
**Name**	**Explains %**	**pseudo-F**	**P**
Tot PAH+$PAH_{alkylated}$	94.5	51.5	0.028
Copper	86.0	18.4	0.082
Zinc	67.1	6.1	0.0745
Lead	54.0	3.5	0.1245
Nickel	40.4	2.0	0.219
Cadmium	29.3	1.2	0.513

**g) Multiple linear regression - % Tail DNA (LYS + net FPG)**			
**Conditional Term Effects:**			
**Name**	**Explains %**	**pseudo-F**	**P**
Tot PAH+$PAH_{alkylated}$	94.50	51.5	0.023
Nickel	4.65	10.8	0.078
Lead	0.71	4.8	0.223
Copper	0.15	<0.1	1.

**d) Linear regression - Nymph length**			
**Simple Term Effects:**			
**Name**	**Explains %**	**pseudo-F**	**P**
Zinc	48.5	2.8	0.1715
Tot PAH+$PAH_{alkylated}$	40.6	2.1	0.238
Copper	25.9	1.1	0.346
Nickel	16.9	0.6	0.446
Cadmium	12.1	0.4	0.524
Lead	0.7	<0.1	0.9135

**h) Multiple linear regression - Nymph length**			
**Conditional Term Effects:**			
**Name**	**Explains %**	**pseudo-F**	**P**
Zinc	48.5	2.8	0.2025
Lead	28.9	2.6	0.253
Copper	19.9	7.5	0.2365
Cadmium	2.7	<0.1	1.

Supplementary Table S 4. Output of the Moran's I test on spatial autocorrelation on the residuals obtained from the statistically significant regression models. Moran's I is a correlation coefficient ranging from -1 (perfect dispersion) to 1 (perfect clustering) that measure the overall spatial autocorrelation in the data. The null hypothesis was that there is no spatial clustering between the residuals and the geographic positions of the ponds in the study area.

**Variable type**		**Moran's Index**			
**Response**	**Predictor**	**Observed**	**Expected**	**Standard Dev.**	**p-value**
**% Tail DNA (LYS)**	**Tot PAH+PAH$_{alkylated}$**	-0.07	-0.25	0.12	0.128
**% Tail DNA (LYS)**	**Zinc**	-0.20	-0.25	0.08	0.552
**% Tail DNA (net FPG)**	**Tot PAH+PAH$_{alkylated}$**	-0.09	-0.25	0.13	0.189
**% Tail DNA (net FPG)**	**Zinc**	-0.18	-0.25	0.11	0.544
**% Tail DNA (LYS + net FPG)**	**Tot PAH+PAH$_{alkylated}$**	-0.26	-0.25	0.11	0.912

Comet assay – Method optimization

The first step in the adaptation of the comet assay to *Aeshna sp.* was the choice of a cell extraction method to obtain a homogenous cell suspension. The hemolymph was chosen as the target cell suspension, as hemocytes are highly sensitive to genotoxic agents, being one of the most commonly used cells for measuring DNA damage in invertebrates including several insect species. Taking in consideration the methods mentioned in literature to extract hemolymph from insects [1], two strategies for hemocyte extraction in dragonfly specimens were performed: dissection of the head and dissection of leg(s) with the help of forceps, after which hemolymph was collected using a microcapillary tube. Several other options at different steps of the general cell extraction were also considered to accommodate specific properties of insect hemolymph. Hemocytes, once isolated, need to be kept in buffer to preserve their viability. However, in the case of insects, the use of anticoagulants is normally required to prevent insect hemolymph from clotting, being phenylthiourea (PTU, CAS number: 103-85-5) one of the most commonly applied [1]. Accordingly, different extraction combinations were tested for dragonfly cell suspensions: hemolymph extracted without addition of buffer, hemolymph extracted and added directly to PBS buffer (without Ca^{2+}/Mg^{2+}, pH 7.4) or PBS buffer with PTU 0.07% (PBS-PTU), and hemolymph extracted to microcapillaries pre-treated with PBS or PBS-PTU. Cell density was also optimized, with different dilutions of the extracted hemolymph tested to ensure enough cell density for a robust scoring of individual cells in the subsequent comet assay: no dilution, 1:1 dilution in PBS-PTU buffer, and cell density adjusted to 2.27×10^5 cells/ml with PBS-PTU buffer.

The optimization experiments to adapt the comet Assay were performed with 10 individuals collected from the Nøstvedt sedimentation pond prior to the sampling campaign described herein. For hemolymph extraction, dragonflies were placed on ice for a few minutes, as gentle cooling is a simple and effective way to anesthetize insects [1]. Once sedated, hemolymph was extracted according to the options above and cell density determined with a Burker counting chamber. Cell density was adjusted with PBS-PTU, after which the trypan blue exclusion assay was used to assess cell viability. All cell suspensions presented a cell viability >95% (data not shown). Samples were then processed according to the method in [2]. An overview of the optimization of the comet assay protocol used for *Aeshna sp.* is represented in Supplementary Figure S *2* 2.

To assess the sensitivity and reproducibility of the selected protocol, the response of cell suspensions (cell density 2.27x10^5 cells/ml) to the positive control hydrogen peroxide (H_2O_2, CAS number: 7722-84-1) was evaluated after 5- and 10-minutes exposure to 50 µM H_2O_2.

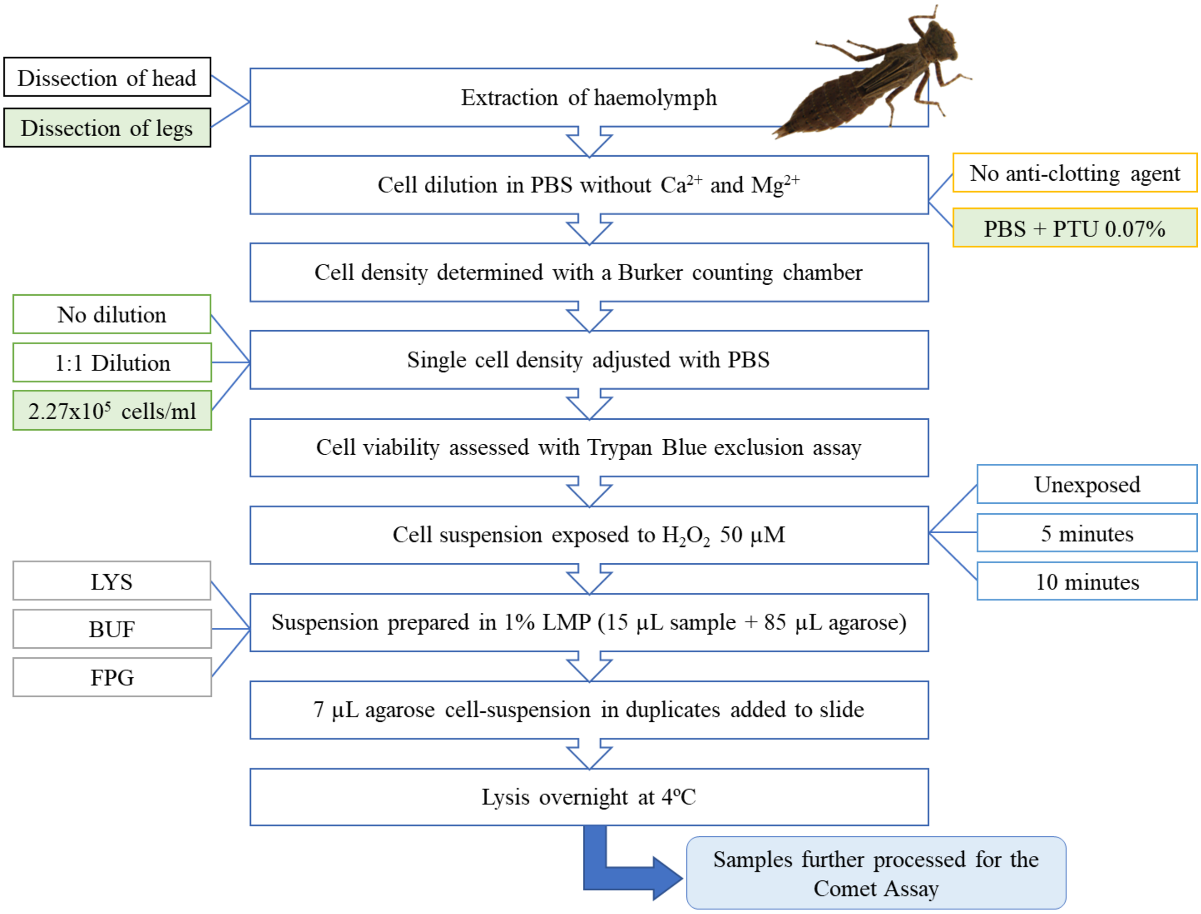

Supplementary Figure S 2. Comet assay protocol adaptation for *Aeshna sp*. Different steps are reported in lateral boxes, with the best option for each step colored in green, whenever relevant. Illustration: Tânia Gomes. Photo of dragonfly nymph: Sondre Meland

Among the different steps of method optimization tested, the optimal yield and quality in terms of cell extraction were obtained by dissecting off the leg and extracting hemolymph with a microcapillary without pre-treatment and directly transferring it to 20µl PBS with 0.07% PTU. As for cell density, visual scoring of the different dilutions showed that a dilution of 2.27x10^5 cells/mL presented a sufficient number of nuclei which were homogenous in dimension. The median values of % tail DNA obtained from the comet assay performed for the different cell density are presented in Supplementary Figure S3. For assessing the sensitivity of the protocol developed, cells were exposed to 50 µM H_2O_2 for 5 and 10 minutes as a positive control. Results obtained show a statistically higher level of % of Tail DNA (LYS) in cells exposed to H_2O_2 for 5 and 10 minutes in comparison with the controls (Supplementary Figure S4). On the other hand, the % Tail DNA measure by net FPG shows an

inverse trend, with higher values being presented by the controls. These results highlight the role of H_2O_2 as a genotoxicant and in particular its capacity to induce single and double strand breaks in cells.

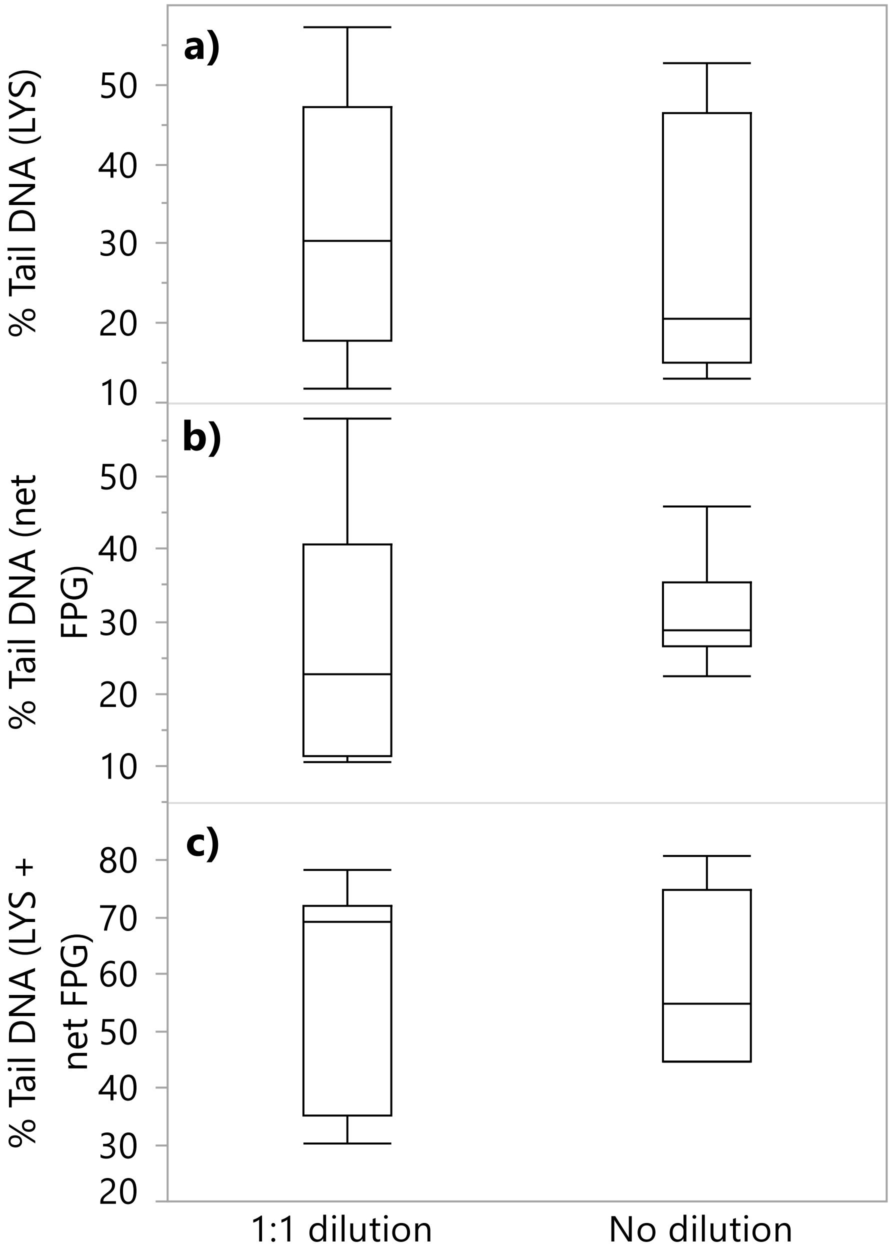

Supplementary Figure S 3. Box-plot showing DNA strand breaks measured in hemolymph cells (no dilution and dilution 1:1) obtained from *Aeshna sp*. in Nøstvedt sedimentation pond. a) % Tail DNA (LYS), b) % Tail DNA (net FPG) and C) % Tail DNA (LYS + net FPG). Individual measurements are included as black points (n = 4).

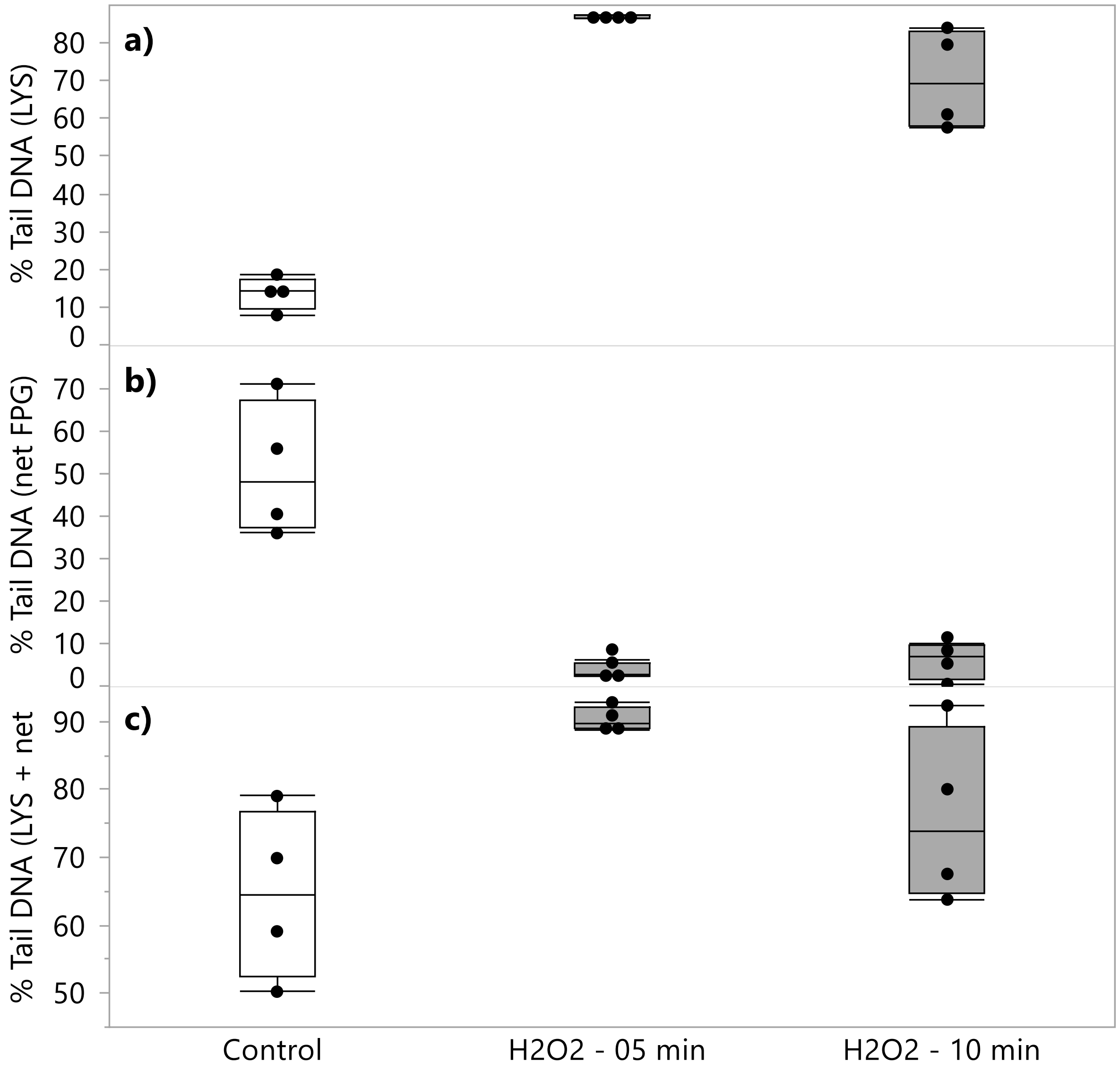

Supplementary Figure S 4. Box-plot showing DNA strand breaks measured in hemolymph cells obtained from *Aeshna sp*. in Nøstvedt sedimentation pond exposed to 50 µM H_2O_2 for 5 and 10 minutes (grey), and unexposed (white). a) % Tail DNA (LYS), b) % Tail DNA (net FPG) and c) % Tail DNA (LYS + net FPG). Individual measurements are included as black points (n = 4).

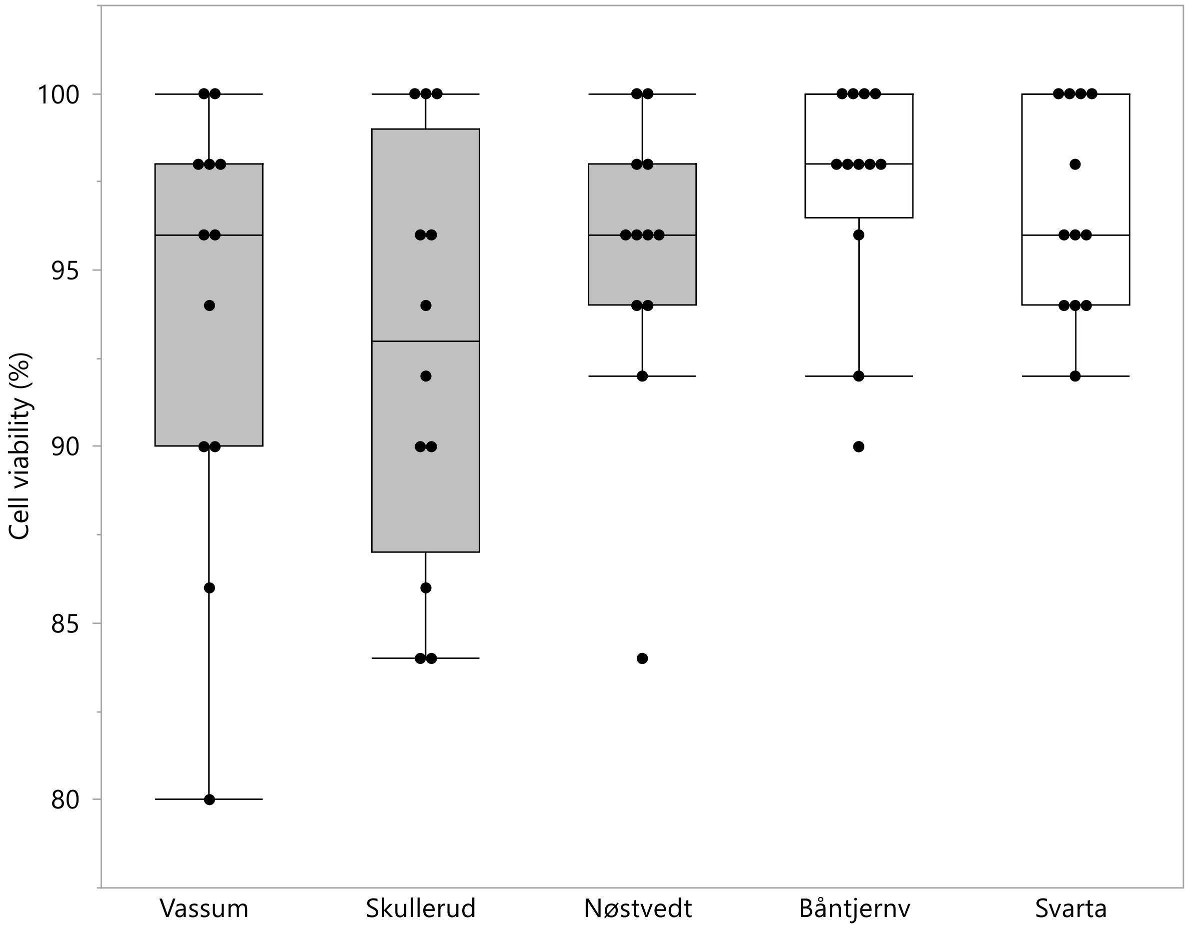

Supplementary Figure S 5. Box-plot showing cell viability using the Trypan Blue exclusion assay measured in dragonfly nymphs (*Aeshna sp.*) living in highway sedimentation ponds (grey) and natural ponds (white). Individual measurements are included as black points (n = 12).

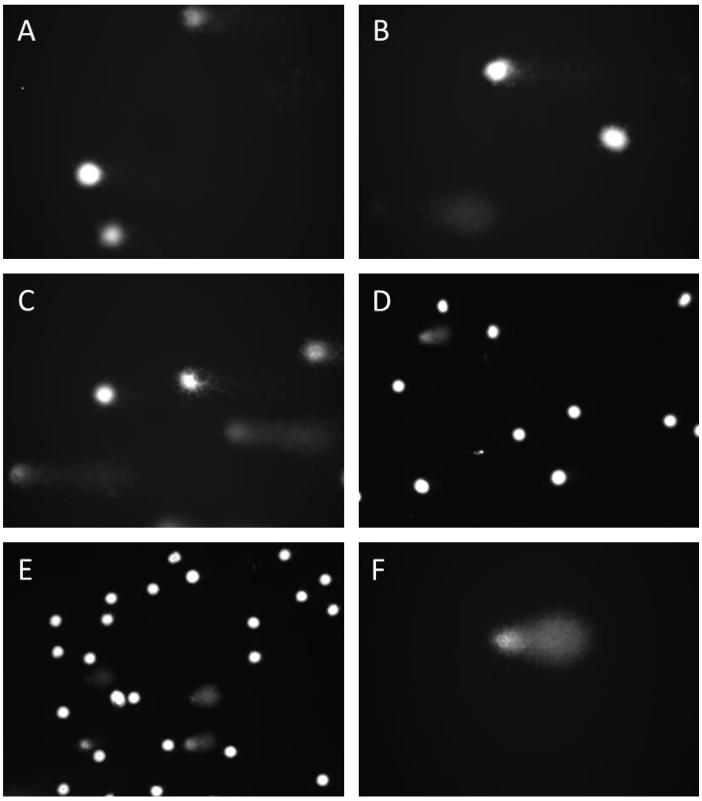

Supplementary Figure S 6. Examples of Aeshna sp. comet assay images, recorded with an optical fluorescence microscope (Olympus BH2-RFL-T3) coupled to a camera (A312f-VIS, BASLER) using a total magnification of x20, showing different examples of (A-E) comet heads (nucleoid core) with no DNA migrating into the tail region, as well as a (F) comet heads (nucleoid core) with broken DNA fragments or damaged DNA migrating away from the nucleus into the tail region.

References

1 Augustyniak, M., Gladysz, M. & Dziewięcka, M. The Comet assay in insects—Status, prospects and benefits for science. *Mutation Research/Reviews in Mutation Research* **767**, 67-76, doi:10.1016/j.mrrev.2015.09.001 (2016).

2 Shaposhnikov, S. *et al.* Twelve-gel slide format optimised for comet assay and fluorescent in situ hybridisation. *Toxicol Lett* **195**, 31-34, doi:10.1016/j.toxlet.2010.02.017 (2010).
